# Associations between antenatal corticosteroids and neonatal morbidities in a prospective cohort: role of course, timing, and gestational age

**DOI:** 10.3389/fped.2026.1784451

**Published:** 2026-03-11

**Authors:** Xuanshu Wang, Kailun Zhang, Xiaomin Ye, Xiwen Wang, Ling Wang, Liya Ma, Hui Liang, Quanfu Zhang, Xu Chen, Ruoqing Chen

**Affiliations:** 1 Department of Epidemiology, School of Public Health (Shenzhen), Sun Yat-sen University, Shenzhen, Guangdong, China; 2 Department of Obstetrics and Gynecology, the Eighth Affiliated Hospital, Sun Yat-sen University, Shenzhen, Guangdong, China; 3 Department of Child Healthcare, Shenzhen Baoan Women’s and Children’s Hospital, Shenzhen, Guangdong, China; 4 Central Laboratory, Shenzhen Baoan Women’s and Children’s Hospital, Shenzhen, Guangdong, China; 5 Department of Obstetrics, Shenzhen Baoan Women’s and Children’s Hospital, Shenzhen, Guangdong, China; 6 Department of Laboratory Medicine, Shenzhen University of Advanced Technology General Hospital, Shenzhen, Guangdong, China

**Keywords:** antenatal corticosteroids, cohort study, dexamethasone, gestational age, neonatal morbidities

## Abstract

**Background:**

Antenatal corticosteroids (ACS) have been widely used to enhance fetal lung maturation in pregnant women at risk of preterm delivery, but gaps remain in understanding how number of courses, gestational age at the first dose, and last dose-to-delivery interval affect neonatal morbidities across different gestational age groups. This study aimed to investigate the associations between ACS, particularly the number of courses, gestational age at the first dose, and last dose-to-delivery interval, and neonatal morbidities.

**Methods:**

This prospective study included 78,642 singleton infants born at 29–43 weeks of gestation between July 2018 and June 2024. Detailed information of ACS exposure and neonatal morbidities was obtained from electronic health records. Logistic regression was applied to estimate the odds ratios (ORs) and 95% confidence intervals (CIs) for neonatal morbidities. Subgroup analyses were performed by stratifying the gestational age at birth.

**Results:**

A total of 2827 (3.59%) infants were exposed to ACS. Compared with unexposed infants, those exposed to ACS had higher risks of respiratory, metabolic, infectious/inflammatory, and neurological morbidities, but not of asphyxia. Multiple ACS courses demonstrated the strongest association with higher risk of neurological morbidity (OR, 2.99; 95% CI 1.68–5.31), along with increased risks of metabolic (OR, 1.43; 95% CI 1.12–1.83) and infectious/inflammatory morbidities (OR, 1.45; 95% CI 1.11–1.89). The timing of the first ACS dose was associated with increased risks of specific neonatal morbidities, regardless of the gestational age at initiation. A last dose-to-delivery interval of 14 days or more was associated with higher risks of metabolic (OR, 1.25; 95% CI 1.14–1.37), infectious/inflammatory (OR, 1.26; 95% CI 1.13–1.40), and neurological (OR, 1.84; 95% CI 1.31–2.59) morbidities. No association was found for infants born before 34 weeks.

**Conclusions:**

ACS exposure, particularly multiple courses or a last dose-to-delivery interval of 14 days or more, was associated with higher risks of neonatal morbidities among infants born at 34 weeks of gestation or later.

## Introduction

Antenatal corticosteroids (ACS), including dexamethasone and betamethasone, are administered to pregnant women at risk of preterm delivery to enhance fetal lung maturation (1). ACS has been demonstrated to significantly reduce the incidence and severity of neonatal respiratory distress syndrome by stimulating surfactant synthesis and secretion in the fetal lung (2–4). Beyond accelerating lung maturation, ACS have also been associated with decreased risks of neonatal death, intraventricular hemorrhage, and other respiratory complications (5–7). However, ACS exposure has been linked to an increased risk of neonatal complications including hypoglycemia, infection, and neurological morbidity (8–11).

Current clinical guidelines consistently recommend ACS for pregnant women between 24^+0^ and 33^+6^ (or 34^+6^) weeks of gestation at risk of preterm delivery within seven days (12–14). However, gaps remain in understanding how the number of courses, gestational age at initial administration, and the interval between treatment and delivery may differentially influence neonatal morbidities. Multiple courses are often administered to women who remain at high risk of preterm delivery after an initial course, yet the benefits and risks of repeated administration remain controversial (15). Some studies reported improved respiratory outcomes, while others suggested possible adverse impact such as impaired fetal growth or neurodevelopmental concerns (16–21). Similarly, the optimal gestational age for the first dose and the ideal treatment-to-delivery interval have not been firmly established. Evidence indicated that an interval of 24 h–7 days is generally associated with maximal benefit. However, some studies have reported benefits beyond this timeframe, while others have observed reduced efficacy or increased risks with prolonged intervals (22, 23). Previous studies have primarily focused on preterm infants, with limited data evaluating how these factors affect outcomes across different gestational age groups.

This study aimed to assess the impact of ACS exposure on neonatal morbidities in infants born at 29–43 weeks of gestation. We specifically examined the number of courses, gestational age at the first dose, and the time interval from last dose to delivery in relation to asphyxia, respiratory, metabolic, infections/inflammatory, and neurological morbidities.

## Materials and methods

This prospective cohort study utilized electronic health records from Shenzhen Baoan Women's and Children's Hospital, one of the two hospitals with the largest number of deliveries in Shenzhen, China (24). Data covering maternal prenatal visits, delivery records, and child health assessments were linked via unique identifiers for each mother-child pair. The eligible population included 106,173 mother-singleton infant dyads from July 2018 to June 2024. As the study focused on neonatal morbidities, infants who did not attend the one-month health check-up at the same hospital (*n* = 27,531) were excluded, resulting in a final analytic sample of 78,624 infants.

ACS were administered as a standard course of four 6-mg intramuscular injections of dexamethasone phosphate at 12 h intervals (25). In clinical practice, variations in ACS exposure occurred due to factors such as the timing of preterm labor, maternal health conditions, and clinical judgment (25–27). We assessed three key exposure variables: the number of courses, gestational age at the first dose, and the interval from the last dose to delivery. The number of courses was categorized as incomplete (<4 doses; *n* = 390), single (4 doses; *n* = 2,144), or multiple (>4 doses; *n* = 293). Among those with multiple courses, 146 had 5–7 doses, 128 had 8 doses, and 19 had more than 8 doses. Gestational age at the first dose, spanning from 24 to 39 weeks, was classified as <34, 34–36, and 37–39 weeks. The last dose-to-delivery interval was analyzed in four categories: <2, 2–7, 8–13, and ≥14 days, as the timing of the last dose is most proximate to delivery and likely to exert the most immediate impact on neonatal outcomes.

The outcomes of interest were defined as neonatal morbidities diagnosed between 0 and 42 days of age. Neonatal morbidities included asphyxia, respiratory morbidity (including respiratory distress syndrome, respiratory failure, meconium aspiration syndrome, transient tachypnea, and others), metabolic morbidity (hypoglycemia and hyperbilirubinemia), infectious/inflammatory morbidity (including sepsis, necrotizing enterocolitis, respiratory tract infections, conjunctivitis, and others), and neurological morbidity (including seizure, intracranial hemorrhage, hypoxic-ischemic encephalopathy, and periventricular leukomalacia). All neonatal morbidities and their corresponding International Classification of Diseases, Tenth Revision (ICD-10) codes are shown in Supplementary Table S1.

Potential confounding variables for the association between ACS administration and neonatal morbidities were identified *a priori* based on clinical expertise and published literature. Maternal characteristics included maternal age at delivery, educational level, parity, mode of conception, pre-pregnancy body mass index (BMI), hypertensive diseases, diabetic diseases, mode of delivery, and vaginal bleeding during early pregnancy (28–31). Infant characteristics included sex, gestational age at birth, birth weight for gestational age percentile, and calendar year of birth (11, 28). Gestational age at birth was determined based on the ultrasound measurement during early pregnancy. Infants were categorized into four groups based on gestational age at birth: <34^+0^ weeks (very and moderate preterm), 34^+0^–36^+6^ weeks (late preterm), 37^+0^–38^+6^ weeks (early term), and >39 weeks (full term, late term, and postterm). Birth weight for gestational age percentiles were calculated according to the national growth standard for newborns by gestational age, and classified into five categories: less than 10th percentile, 10th–24th percentile, 25th–74th percentile, 75th–89th percentile, and greater than or equal to 90th percentile (32). Information about ICD-10 codes for maternal diseases are shown in Supplementary Table S1.

Maternal and infant characteristics were described using mean with standard deviation (SD) or median with interquartile range (IQR) for continuous variables and frequency with percentage for categorical variables. Group comparisons were performed using t-tests/ANOVA or Mann–Whitney *U*/Kruskal–Wallis tests for normally and non-normally distributed continuous variables, and chi-squared or Fisher's exact tests for categorical variables.

Logistic regression was applied to examine the association of ACS exposure, number of courses, gestational age at the first dose, and the last dose-to-delivery interval with neonatal morbidities. Several covariates had missing data: maternal educational level (5.36%), parity (0.01%), mode of conception (3.55%), pre-pregnancy BMI (3.83%), and vaginal bleeding during early pregnancy (21.44%). To address these missing values, multiple imputation by chained equations was performed, generating 10 imputed datasets with 50 iterations each. The multivariable models were adjusted for all aforementioned covariates after multiple imputation. We conducted stratified analyses to estimate odds ratios (ORs) for neonatal morbidities by gestational age at birth and infant sex, including formal tests of interaction to evaluate effect modification. To account for correlations among infants of the same mother, cluster-robust standard errors were implemented. Statistical analyses were performed using Stata/MP version 17 (StataCorp, College Station, TX, USA) and R version 4.4.1 (R Foundation for Statistical Computing, Vienna, Austria).

## Results

We included 78,642 infants in the final analysis. Compared with the excluded infants (*n* = 27,531), the included population had a higher proportion of term births and was more likely to have mothers with the following characteristics: higher education, primiparity, and lower proportions of assisted conception, hypertensive diseases, vaginal bleeding during early pregnancy, and cesarean delivery (Supplementary Table S2). A total of 2,827 (3.59%) infants were exposed to ACS (Table 1). The proportions of exposed infants by gestational age at birth were 3.43% (*n* = 97, 29^+0^–33^+6^ weeks), 24.27% (*n* = 686, 34^+0^–36^+6^ weeks), 38.56% (*n* = 1,090, 37^+0^–38^+6^ weeks), 31,73% (*n* = 897, 39^+0^–40^+6^ weeks), and 2.02% (*n* = 57, 41^+0^–41^+6^ weeks). Compared with unexposed infants, those exposed to ACS were more likely to have mothers with the following characteristics: older at delivery, primiparity, assisted conception, pre-pregnancy underweight or overweight, diabetic or hypertensive diseases, vaginal bleeding during early pregnancy, and cesarean delivery. The distribution of characteristics according to the number of courses, gestational age at the first dose, and last dose-to-delivery interval are shown in Supplementary Tables S3–S5.

**Table 1:** Characteristics of the study population according to ACS exposure.

Characteristics	Overall [*n* (%)]	Unexposed to ACS [*n* (%)]	Exposed to ACS [*n* (%)]	P value^b^
Total^a^	78,642 (100.00)	75,815 (96.41)	2,827 (3.59)	
Mothers				
Age at delivery				
As continuous variable [mean (SD)]	30.38 (4.07)	30.36 (4.06)	30.82 (4.46)	<0.001
<25	4,912 (6.25)	4,737 (6.25)	175 (6.19)	<0.001
25–29	29,477 (37.48)	28,518 (37.62)	959 (33.92)	
30–34	32,101 (40.82)	30,958 (40.83)	1,143 (40.43)	
≥35	12,152 (15.45)	11,602 (15.30)	550 (19.46)	
Educational level				
Junior high school or below	6,028 (7.67)	5,777 (7.62)	251 (8.88)	<0.001
Senior high school or vocational school	11,193 (14.23)	10,802 (14.25)	391 (13.83)	
University or above	57,204 (72.74)	55,231 (72.85)	1,973 (69.79)	
Missing	4,217 (5.36)	4,005 (5.28)	212 (7.50)	
Parity				
1	42,535 (54.09)	40,928 (53.98)	1,607 (56.84)	<0.001
2	30,862 (39.24)	29,856 (39.38)	1,006 (35.59)	
≥3	5,241 (6.66)	5,027 (6.63)	214 (7.57)	
Missing	4 (0.01)	4 (0.01)	0 (0.00)	
Mode of conception				
Naturally conceived	72,819 (92.60)	70,371 (92.82)	2,448 (86.59)	<0.001
ART	3,031 (3.85)	2,811 (3.71)	220 (7.78)	
Missing	2,792 (3.55)	2,633 (3.47)	159 (5.62)	
Pre-pregnancy BMI				
As continuous variable [median (IQR)]	20.58 (19.05, 22.55)	20.57 (19.07, 22.55)	20.63 (18.90, 22.86)	0.691
<18.5 (underweight)	13,113 (16.67)	12,598 (16.62)	515 (18.22)	<0.001
18.5–23.9 (normal weight)	52,085 (66.23)	50,380 (66.45)	1,705 (60.31)	
24.0–27.9 (overweight)	8,660 (11.01)	8,298 (10.95)	362 (12.81)	
≥28.0 (obesity)	1,772 (2.25)	1,690 (2.23)	82 (2.90)	
Missing	3,012 (3.83)	2,849 (3.76)	163 (5.77)	
Diabetic disease				
No	62,903 (79.99)	60,825 (80.23)	2,078 (73.51)	<0.001
Yes	15,739 (20.01)	14,990 (19.77)	749 (26.49)	
Hypertensive disease				
No	73,864 (93.92)	71,357 (94.12)	2,507 (88.68)	<0.001
Yes	4,778 (6.08)	4,458 (5.88)	320 (11.32)	
Vaginal bleeding during early pregnancy				
No	50,482 (64.19)	48,901 (64.50)	1,581 (55.93)	<0.001
Yes	11,302 (14.37)	10,719 (14.14)	583 (20.62)	
Missing	16,858 (21.44)	16,195 (21.36)	663 (23.45)	
Mode of delivery				
Vaginal delivery	51,436 (65.41)	49,941 (65.87)	1,495 (52.88)	<0.001
Elective cesarean section	15,737 (20.01)	14,953 (19.72)	784 (27.73)	
Emergency cesarean section	11,469 (14.58)	10,921 (14.40)	548 (19.38)	
Infants				
Sex				
Male	41,927 (53.31)	40,380 (53.26)	1,547 (54.72)	0.131
Female	36,715 (46.69)	35,435 (46.74)	1,280 (45.28)	
Gestational age at birth (weeks)				
As continuous variable [median (IQR)]	39.43 (38.71, 40.14)	39.43 (38.71, 40.14)	38.14 (36.71, 39.29)	<0.001
<34	143 (0.18)	46 (0.06)	97 (3.43)	<0.001
34–36	2,015 (2.56)	1,329 (1.75)	686 (24.27)	
37–38	22,669 (28.83)	21,579 (28.46)	1,090 (38.56)	
39–40	48,057 (61.11)	47,160 (62.20)	897 (31.73)	
≥41	5,758 (7.32)	5,701 (7.52)	57 (2.02)	
Birth weight for gestational age (percentile)				
<10th	6,303 (8.01)	6,073 (8.01)	230 (8.14)	0.265
10th–24th	12,055 (15.33)	11,664 (15.38)	391 (13.83)	
25th–74th	40,452 (51.44)	38,979 (51.41)	1,473 (52.10)	
75th–89th	12,026 (15.29)	11,579 (15.27)	447 (15.81)	
≥90th	7,806 (9.93)	7,520 (9.92)	286 (10.12)	
Calendar year of birth				
2018–2019	21,976 (27.94)	21,289 (28.08)	687 (24.30)	<0.001
2020	13,133 (16.70)	12,762 (16.83)	371 (13.12)	
2021	13,554 (17.24)	13,025 (17.18)	529 (18.71)	
2022	12,423 (15.80)	11,903 (15.70)	520 (18.39)	
2023–2024	17,556 (22.32)	16,836 (22.21)	720 (25.47)	

ART, assisted reproductive technology; BMI, body mass index; IQR, interquartile range; SD, standard deviation.

a Number and row percentage.

b Continuous variables were compared using t test or Mann–Whitney U test; categorical variables were compared using chi-squared test.

**Figure 1:**
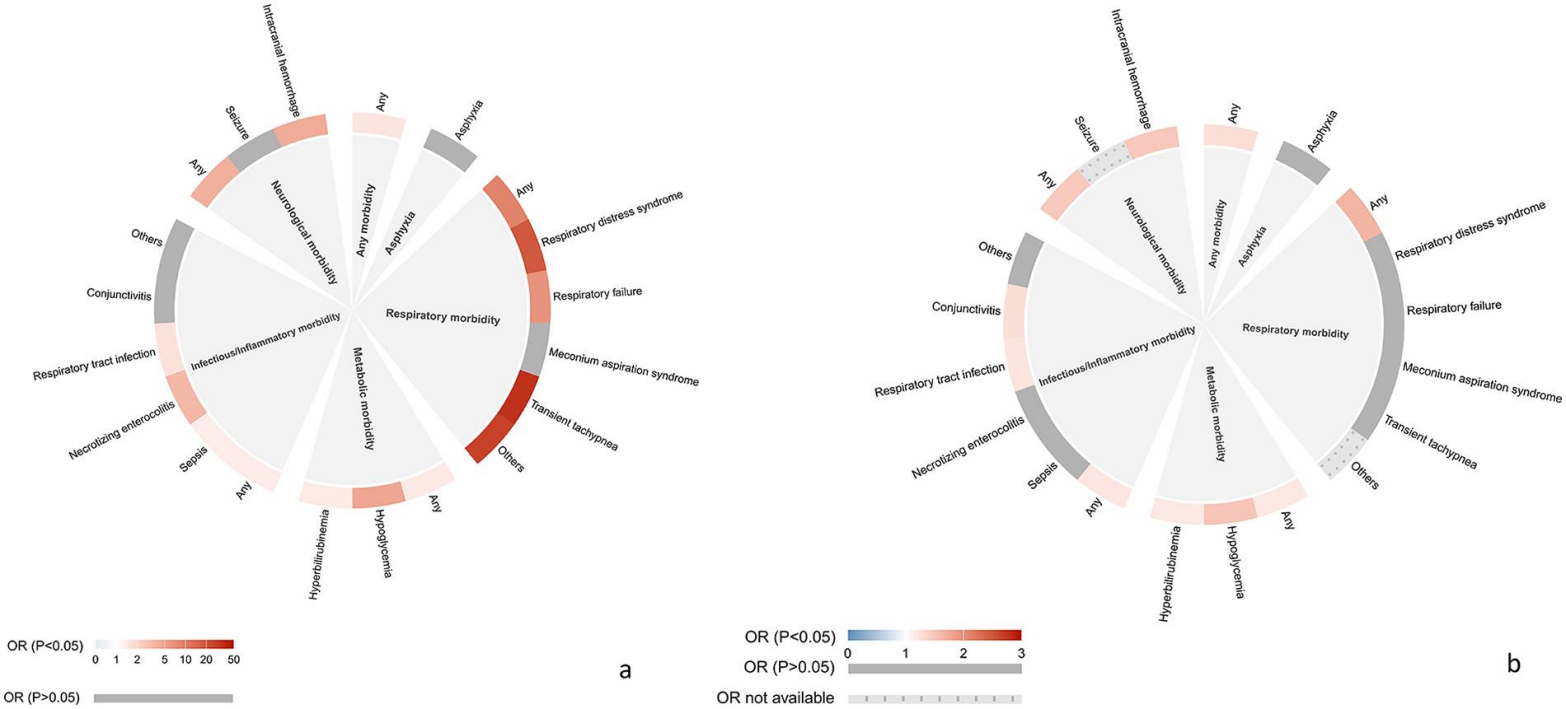
Associations between antenatal corticosteroid (ACS) exposure and neonatal morbidities. Plot a presents the odds ratios from the crude model, and plot b shows the odds ratios from the model adjusted for maternal age at delivery, educational level, parity, mode of conception, pre-pregnancy BMI, hypertensive diseases, diabetic diseases, mode of delivery, and vaginal bleeding during early pregnancy, as well as infant sex, gestational age at birth, birth weight for gestational age (percentile), and calendar year of birth, with all covariates handled using multiple imputation by chained equations. Point estimates are displayed for each neonatal morbidity outcome. Colored markers indicate statistically significant associations (*P* < 0.05). Grey markers indicate non-significant results. Grey markers with dotted patterns indicate results where ORs were not available due to non-convergence of the model caused by the limited number of cases. The degree of color represents the magnitude of the corresponding association. Detailed results are provided in Supplementary Table S6.

**Figure 2:**
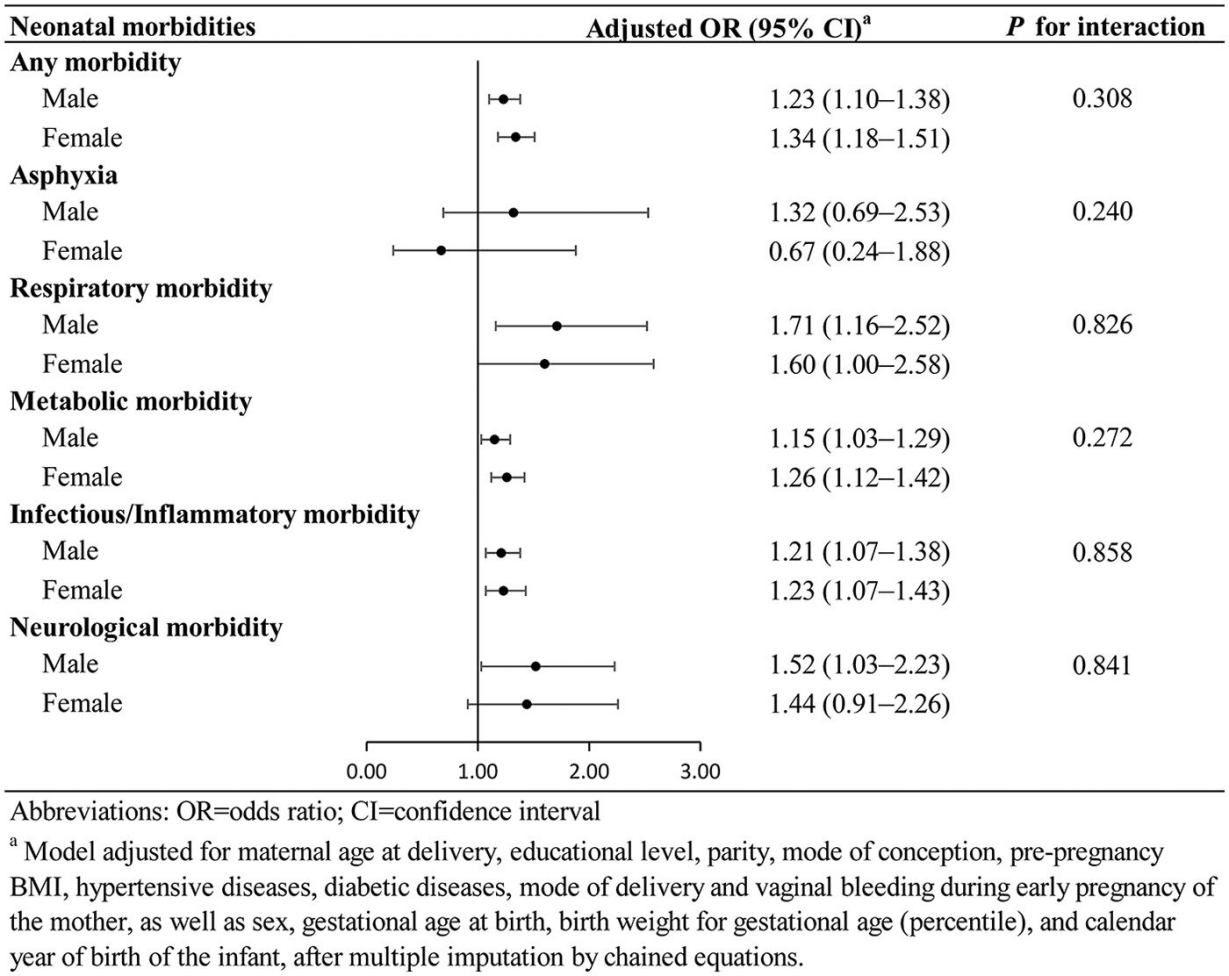
Association between ACS exposure and neonatal morbidities stratified by sex. This figure shows odds ratios and 95% confidence intervals for neonatal morbidities comparing ACS-exposed versus unexposed infants, stratified by infant sex.

Among infants unexposed to ACS (*n* = 75,815), approximately 51.0% (*n* = 38,659) were diagnosed with any type of neonatal morbidity. However, among infants exposed to ACS (*n* = 2,827), the proportion with any neonatal morbidity was 62.1% (*n* = 1,756). Compared with unexposed infants, those exposed to ACS showed an increased overall risk of any neonatal morbidity (OR: 1.28, 95% CI: 1.17–1.39). Specifically, elevated risks were observed for respiratory, metabolic (including hypoglycemia and hyperbilirubinemia), infectious/inflammatory (notably respiratory tract infection and conjunctivitis), and neurological morbidities, though no association was found with asphyxia (Figure 1; Table 2). Sex-stratified analyses revealed that respiratory morbidity, metabolic morbidity and infectious/inflammatory morbidity were increased in both sexes. However, elevated risk of neurological morbidity was specific to male exposed infants (Figure 2).

**Table 2:** Association between ACS exposure and neonatal morbidities.

Neonatal morbidities	Unexposed to ACS (*n* = 75,815)		Exposed to ACS (*n* = 2,827)		
	*N* of cases (rate)^a^	OR (95% CI)	*N* of cases (rate)^a^	Crude OR (95% CI)	Adjusted OR (95% CI)^b^
Any morbidity	38,659 (509.91)	Ref.	1,756 (621.15)	1.58 (1.46–1.70)	1.28 (1.17–1.39)
Asphyxia	343 (4.52)	Ref.	20 (7.07)	1.57 (1.00–2.46)	1.10 (0.61–1.96)
Respiratory morbidity	275 (3.63)	Ref.	88 (31.13)	8.83 (6.92–11.25)	1.67 (1.21–2.31)
Respiratory distress syndrome	80 (1.06)	Ref.	56 (19.81)	18.57 (13.07–26.38)	1.43 (0.92–2.20)
Respiratory failure	118 (1.56)	Ref.	29 (10.26)	6.66 (4.43–10.01)	1.29 (0.73–2.26)
Meconium aspiration syndrome	98 (1.29)	Ref.	3 (1.06)	0.82 (0.26–2.59)	1.18 (0.33–4.20)
Transient tachypnea	13 (0.17)	Ref.	16 (5.66)	33.22 (15.97–69.14)	2.72 (0.97–7.64)
Others	1 (0.01)	Ref.	0 (0)	26.85 (5.42–133.07)	NA
Metabolic morbidity	33,440 (441.07)	Ref.	1,511 (534.49)	1.45 (1.35–1.57)	1.20 (1.11–1.31)
Hypoglycemia	33,305 (439.29)	Ref.	1,489 (526.71)	4.73 (3.47–6.44)	1.51 (1.06–2.16)
Hyperbilirubinemia	276 (3.64)	Ref.	48 (16.98)	1.42 (1.32–1.53)	1.19 (1.09–1.29)
Infectious/Inflammatory morbidity	12,931 (170.56)	Ref.	628 (222.14)	1.39 (1.27–1.52)	1.22 (1.11–1.35)
Sepsis	1,466 (19.34)	Ref.	74 (26.18)	1.36 (1.08–1.73)	1.24 (0.96–1.61)
Necrotizing enterocolitis	198 (2.61)	Ref.	25 (8.84)	3.41 (2.24–5.17)	1.42 (0.85–2.39)
Respiratory tract infection	6,367 (83.98)	Ref.	371 (131.23)	1.65 (1.47–1.84)	1.23 (1.09–1.40)
Conjunctivitis	2,859 (37.71)	Ref.	123 (43.51)	1.16 (0.97–1.40)	1.28 (1.05–1.56)
Others	2,965 (39.11)	Ref.	105 (37.14)	0.95 (0.78–1.16)	1.10 (0.89–1.35)
Neurological morbidity	450 (5.94)	Ref.	66 (23.35)	4.00 (3.08–5.20)	1.48 (1.08–2.04)
Seizure	45 (0.59)	Ref.	2 (0.71)	1.19 (0.29–4.92)	NA
Intracranial hemorrhage	401 (5.29)	Ref.	64 (22.64)	4.36 (3.34–5.69)	1.49 (1.08–2.07)
Hypoxic-ischemic encephalopathy	7 (0.09)	Ref.	0 (0)	NA	NA
Periventricular leukomalacia	1 (0.01)	Ref.	0 (0)	NA	NA

OR, odds ratio; CI, confidence interval; Na, Nnot available due to convergence not achieved caused by the limited number of cases.

a Rate represents the number of cases per thousand infants.

b Model adjusted for maternal age at delivery, educational level, parity, mode of conception, pre-pregnancy BMI, hypertensive diseases, diabetic diseases, mode of delivery and vaginal bleeding during early pregnancy of the mother, as well as sex, gestational age at birth, birth weight for gestational age (percentile), and calendar year of birth of the infant, after multiple imputation by chained equations.

The association between ACS exposure and neonatal morbidities varied by the number of courses administered. While an incomplete course was primarily associated with higher risk of respiratory morbidity (OR: 1.89, 95% CI: 1.15–3.10), a single course was also associated with higher risks of respiratory, metabolic, and infectious/inflammatory morbidities. Multiple courses were associated with even higher risks of metabolic and infectious/inflammatory morbidities, with the strongest association observed for neurological morbidity (OR: 2.99, 95% CI: 1.68–5.31). Exposure to the initial ACS dose adversely affected neonatal morbidities across all gestational ages, though the specific morbidities affected varied with timing. The risks of respiratory and metabolic morbidities increased when the first dose was administered at 34–36 weeks, and became even higher when administered at 37–39 weeks. On the contrary, the risk of infectious/inflammatory morbidity was elevated with earlier administration. Notably, the increased risk of neurological morbidity was confined to initial administration before 34 weeks (OR: 1.98, 95% CI: 1.39–2.82). Associations were observed between the last dose-to-delivery interval and respiratory morbidity, with increased risks at intervals <8 days and ≥14 days. An interval of ≥14 days was also associated with higher risks of metabolic, infectious/inflammatory, and neurological morbidities. No association was observed for intervals of 8–13 days (Table 3).

**Table 3:** Associations of number of courses, gestational age at the first dose (weeks), and last dose-to-delivery interval (days) with neonatal morbidities.

Neonatal morbidities	Unexposed to ACS (*n* = 75,815)	Exposed to ACS (*n* = 2,827)									
		Number of courses			Gestational age at the first dose (weeks)			Last dose-to-delivery interval (days)			
		Incomplete course	Single course	Multiple courses	<34	34–36	37–39	<2	2–7	8–13	≥14
Any morbidity											
*N* of cases (rate)^a^	38,659 (509.91)	271 (694.87)	1,278 (596.08)	207 (706.48)	1,082 (620.41)	654 (622.26)	20 (625.00)	183 (104.93)	226 (215.03)	92 (2,875.00)	1,255 (16.55)
OR (95% CI)^b^	Ref.	1.34 (1.06–1.69)	1.24 (1.13–1.36)	1.62 (1.25–2.10)	1.31 (1.18–1.45)	1.21 (1.06–1.38)	1.76 (0.87–3.54)	1.14 (0.84–1.54)	1.09 (0.85–1.41)	1.11 (0.79–1.56)	1.32 (1.20–1.45)
Asphyxia											
*N* of cases (rate)a	343 (4.52)	3 (7.69)	14 (6.53)	3 (10.24)	16 (9.17)	3 (2.85)	1 (31.25)	2 (1.15)	2 (1.90)	2 (62.50)	14 (0.18)
OR (95% CI)^b^	Ref.	0.78 (0.24–2.55)	1.16 (0.61–2.18)	1.24 (0.35–4.34)	1.37 (0.72–2.60)	0.46 (0.14–1.59)	9.21 (1.16–73.36)	0.50 (0.13–1.98)	0.36 (0.07–1.96)	2.03 (0.52–7.91)	1.40 (0.79–2.47)
Respiratory morbidity											
*N* of cases (rate)a	275 (3.63)	26 (66.67)	48 (22.39)	14 (47.78)	56 (32.11)	31 (29.50)	1 (31.25)	23 (13.19)	32 (30.45)	6 (187.50)	27 (0.36)
OR (95% CI) ^b^	Ref.	1.89 (1.15–3.10)	1.64 (1.12–2.40)	1.43 (0.72–2.83)	1.48 (0.97–2.26)	1.84 (1.18–2.88)	9.64 (1.41–65.86)	1.79 (1.05–3.07)	1.73 (1.01–2.94)	1.62 (0.67–3.91)	1.59 (1.03–2.45)
Metabolic morbidity											
*N* of cases (rate)a	33,440 (441.07)	234 (600.00)	1,099 (512.59)	178 (607.51)	919 (526.95)	572 (544.24)	20 (625.00)	154 (88.30)	189 (179.83)	79 (2,468.75)	1,089 (14.36)
OR (95% CI)^b^	Ref.	1.24 (0.99–1.55)	1.17 (1.07–1.29)	1.43 (1.12–1.83)	1.21 (1.10–1.34)	1.16 (1.01–1.32)	2.28 (1.13–4.61)	1.00 (0.75–1.32)	1.02 (0.80–1.30)	1.04 (0.74–1.45)	1.25 (1.14–1.37)
Infectious/Inflammatory morbidity											
N of cases (rate)a	12,931 (170.56)	94 (241.03)	455 (212.22)	79 (269.62)	398 (228.21)	227 (215.98)	3 (93.75)	67 (38.42)	83 (78.97)	33 (1,031.25)	445 (5.87)
OR (95% CI)^b^	Ref.	1.15 (0.90–1.47)	1.21 (1.08–1.35)	1.45 (1.11–1.89)	1.25 (1.11–1.41)	1.20 (1.02–1.40)	0.59 (0.18–1.88)	1.11 (0.82–1.50)	1.08 (0.81–1.43)	1.17 (0.79–1.74)	1.26 (1.13–1.40)
Neurological morbidity											
*N* of cases (rate)a	450 (5.94)	15 (38.46)	33 (15.39)	18 (61.43)	45 (25.80)	20 (19.03)	1 (31.25)	11 (6.31)	14 (13.32)	2 (62.50)	39 (0.51)
OR (95% CI)b	Ref.	1.38 (0.74–2.55)	1.24 (0.85–1.82)	2.99 (1.68–5.31)	1.98 (1.39–2.82)	0.89 (0.53–1.47)	4.81 (0.62–37.66)	1.01 (0.51–2.01)	1.18 (0.63–2.22)	0.62 (0.15–2.56)	1.84 (1.31–2.59)

OR, odds ratio; CI, confidence interval; NA, not available.

a Rate represents the number of cases per thousand children.

b Model adjusted for maternal age at delivery, educational level, parity, mode of conception, pre-pregnancy BMI, hypertensive diseases, diabetic diseases, mode of delivery and vaginal bleeding during early pregnancy of the mother, as well as sex, gestational age at birth, birth weight for gestational age (percentile), and calendar year of birth of the infant, after multiple imputation by chained equations.

**Figure 3:**
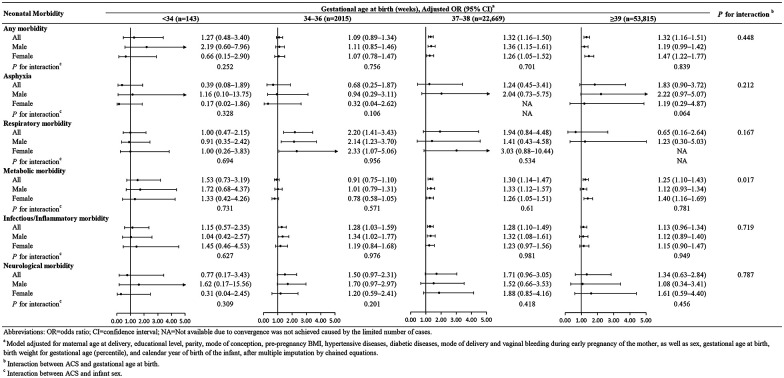
Association between ACS exposure and neonatal morbidities stratified by gestational age. This figure shows odds ratios and 95% confidence intervals for neonatal morbidities comparing ACS-exposed versus unexposed infants, stratified by gestational age at birth and infant sex.

Stratification by gestational age at birth revealed distinct associations between ACS exposure and neonatal morbidities. No association was found in infants born before 34 weeks. Among infants born at 34–36 weeks, ACS exposure was associated with higher risks of respiratory (OR: 2.20, 95% CI: 1.41–3.43) and infectious/inflammatory (OR: 1.28, 95% CI: 1.03–1.59) morbidities. In infants delivered at 37–38 weeks, ACS was associated with elevated risks of metabolic (OR: 1.30, 95% CI: 1.14–1.47) and infectious/inflammatory morbidities (OR: 1.28, 95% CI: 1.10–1.49). Among those born at 39 weeks or later, ACS exposure was associated with an increased risk of metabolic morbidity, particularly in female infants (OR: 1.40, 95% CI: 1.16–1.69). However, interaction effects between ACS and gestational age at birth or sex of the infant were largely not observed (Figure 3).

Further classification of ACS exposure revealed that among infants born at 34–36 weeks, only those exposed to an incomplete course had a higher risk of respiratory morbidity. Infants born at 37–38 weeks who were exposed to an incomplete or single course showed heightened risks of metabolic and infectious/inflammatory morbidities. Infants born at ≥39 weeks and exposed to a single course had also a higher risk of metabolic morbidity. Moreover, infants born at ≥34 weeks who were exposed to multiple courses had an increased risk of neurological morbidity (Supplementary Table S6).

Furthermore, among infants born at 34–36 weeks, initial ACS administration before 34 weeks was associated with higher risks of respiratory, infectious/inflammatory, and neurological morbidities. For infants born at 37–38 weeks, initial exposure before 37 weeks rendered higher risks of neonatal morbidities. Similarly, among infants born at ≥39 weeks, initial ACS before 37 weeks was associated with a higher risk of metabolic morbidity (Supplementary Table S7).

A last dose-to-delivery interval of <8 days was associated with a higher risk of respiratory morbidity among infants born at 34–36 weeks, whereas an interval of ≥14 days was associated with a higher risk of neurological morbidity in the same group. Similar associations were observed for metabolic and infectious/inflammatory morbidities among infants born at ≥37 weeks (Supplementary Table S8).

## Discussion

This study found that ACS exposure, particularly multiple courses or a last dose-to-delivery interval of 14 days or more, was associated with higher risks of neonatal respiratory, metabolic, infectious/inflammatory, and neurological morbidities among infants. However, no association was found between ACS exposure and risk of asphyxia. Furthermore, the first ACS administration, regardless of the gestational age at initiation, was associated with increased risks of specific neonatal morbidities. No association between ACS exposure and neonatal morbidity was found in infants born before 34 weeks of gestation.

Exposure to multiple ACS courses was associated with higher risks of neonatal morbidities, with the strongest association observed for neurological morbidity, and more moderate associations for metabolic and infectious/inflammatory outcomes. This pattern suggests that neurological outcomes may be particularly sensitive to repeated ACS exposure. These findings are consistent with previous studies reporting a higher rate of intraventricular hemorrhage, leukomalacia, and cerebral palsy among infants exposed to multiple courses of ACS (10, 33–35). Some animal studies also reported detrimental effects of multiple courses of ACS on fetal neural development (36, 37). The underlying mechanism may involve elevated glucocorticoid levels, which can induce lasting alterations in the hypothalamic-pituitary-adrenal (HPA) axis and impede brain development, thereby increasing vulnerability to neonatal neurological morbidity (38, 39). We also observed that infants exposed to multiple ACS courses had higher risks of metabolic and infectious/inflammatory morbidities, although the magnitude of these associations was comparatively moderate. Repeated glucocorticoid exposure has been shown to induce insulin resistance and impair glucose metabolism in the fetus, increasing the risk of postnatal metabolic disturbances (40–42). Moreover, given the well-established immunosuppressive effects of glucocorticoids, multiple ACS courses may compromise fetal immune function, increasing susceptibility to infectious and inflammatory conditions (43). In contrast, an incomplete course of ACS was associated with a higher risk of respiratory morbidity particularly among infants born at 34–36 weeks, consistent with findings from an earlier Italian study (44). This may reflect insufficient glucocorticoids exposure to adequately promote fetal lung maturation, resulting in compromised respiratory function after birth.

Timing of the initial ACS dose was found to differentially influence neonatal morbidities, with later administration associated with elevated risks of respiratory and metabolic morbidities. This may be because administration at a later gestational age misses the critical window for pulmonary maturation. Such timing fails to elicit a sufficient maturational response in the fetal lungs, thereby predisposing the newborn to respiratory morbidity (45). Furthermore, additional exogenous glucocorticoids during this period may disrupt metabolic homeostasis and increase vulnerability to metabolic morbidities. In contrast, earlier initiation of ACS was associated with a higher risk of neurological morbidity, likely because it coincides with the susceptible period of early fetal brain development, potentially interfering with fundamental processes including neuronal proliferation, migration, and differentiation (46, 47).

In this study, we observed that a last dose-to-delivery interval of 14 days or more was associated with increased risk of respiratory, metabolic, infectious/inflammatory, and neurological morbidities among infants. Similarly, a previous US study also found that ACS exposure ≥14 days before delivery was associated with increased risks of severe neonatal morbidity, including death, severe intraventricular hemorrhage, periventricular leukomalacia, bronchopulmonary dysplasia, and necrotizing enterocolitis (48). Mechanistically, this may reflect: 1) waning of the steroid-induced stimulus for surfactant production, leaving the newborn unprepared for extrauterine respiratory demands; 2) premature steroid exposure may downregulate glucocorticoid receptor expression or sensitivity in fetal tissues, impairing the infant's adaptive stress response during birth; 3) steroid-induced programming of the fetal HPA axis and metabolism, along with thymic and immune alterations, may further increase the susceptibility to metabolic, infectious/inflammatory, and neurological morbidities (48–50). Although previous evidence suggests a protective effect of ACS exposure within 7 days before birth against respiratory distress syndrome for late preterm infants (51), our findings show that a last dose-to-delivery interval of 7 days or less was associated with an increased risk of respiratory morbidity primarily among infants born at 34–36 weeks of gestation. Potential explanations include transient suppression of the fetal stress response by recent glucocorticoid exposure, which impairs cardiorespiratory adaption at birth, thereby compromising postnatal respiratory function (52).

ACS administration has been recommended for pregnancies before 33^+6^ (or 34^+6^) weeks of gestation at risk of preterm delivery, given its beneficial effects in reducing respiratory complications and mortality in neonates (3). In this study, no evidence was found for an association between ACS exposure and any neonatal morbidity among infants born before 34 weeks. In contrast, ACS exposure in late preterm infants (34–36 weeks) conferred a modest elevation in risk for respiratory and infectious/inflammatory morbidities. This is consistent with earlier reports suggesting potential harm of ACS in this subgroup (53, 54). Around 34–36 weeks of gestation, the respiratory benefits of ACS diminish as fetal lung maturation advances (55, 56). Exogenous corticosteroids at this stage may potentially disrupt hormonal balance, which could partially offset respiratory benefits and contribute to a higher respiratory risk (56). In addition, reduced placental protection due to the low affinity of synthetic steroids such as betamethasone and dexamethasone for 11β-hydroxysteroid dehydrogenase type 2 allows greater transplacental transfer of these agents, which could suppress fetal immune function and heighten susceptibility to infection or inflammation during this gestational window (49, 57). We further observed that among infants born at 37 gestational weeks or later, ACS exposure was associated with increased risks of metabolic and infectious/inflammatory morbidities. These associations were characterized by smaller effect sizes, suggesting limited but potentially clinically relevant increases in risk at the population level. Similar observations have been reported previously, with dexamethasone exposure linked to higher risks of neonatal morbidities such as hypoglycemia, hyperbilirubinemia, and sepsis among term-born infants (58). One possible explanation is that exogenous glucocorticoids exposure in late gestation may act synergistically with the physiologic cortisol surge near term, leading to an additive glucocorticoid burden that predisposes newborns to these complications (39, 59).

Previous evidence suggests that the impact of ACS may differ by infant sex. For instance, some studies found that ACS-exposed male infants had a higher incidence of bronchopulmonary dysplasia, intraventricular hemorrhage (grade III-IV), and a longer median duration of mechanical ventilation days than ACS-exposed female infants (60, 61). However, our study did not find a sex-specific difference in ACS-related neonatal morbidities. The discrepancy may stem from variations in population characteristics across studies, as well as potential differences in sex-related gene expression that may modulate the effect of ACS in different populations.

Our findings underscore two critical considerations for ACS administration in clinical practice. First, a more evidence-based approach is warranted regarding repeated courses and the timing of the initial dose, as these factors profoundly influence the risk of neonatal morbidities. Second, infants exposed to ACS, particularly those born at or beyond 34 weeks of gestation or with a last dose-to-delivery interval exceeding 14 days, may benefit from enhanced surveillance to facilitate the early detection and management of potential complications.

Building on these clinical considerations, our study also identifies several key gaps that should be addressed by subsequent investigation. First, while our findings elucidate the association between ACS timing and neonatal morbidity, the long-term health outcomes of exposed infants across different gestational ages warrant further investigation. Mechanistic studies are also needed to delineate the biological pathways underlying the differential effects observed with varying ACS exposure windows. Furthermore, well-designed randomized controlled trials are essential to evaluate the impacts of specific ACS protocols—including the number of courses, timing of the initial dose, and the dose-to-delivery interval—on neonatal health.

This study has several notable strengths, including its prospective hospital-based design, large population, and comprehensive data collection, which allowed for a detailed examination of ACS exposure and neonatal morbidities. A key feature is the fine-grained categorization of exposed groups, particularly with respect to the gestational age at the first ACS dose, a variable seldom explored in prior research. Moreover, the study's scope encompassed a broad spectrum of neonatal morbidities and included infants across a wide range of gestational ages at birth, thereby allowing a holistic analysis of associations between different exposure categories and neonatal morbidities in various gestational age groups. However, the study also has some limitations. First, while extensive adjustments were made for maternal and infant characteristics, the potential for residual confounding from unmeasured factors cannot be entirely ruled out. Second, the included study population differed from the excluded population in several maternal characteristics, which may affect the representativeness of the results. Third, although ACS is typically administered based on clinical assessment of threatened preterm delivery in routine practice, detailed information on the specific indications or decision-making processes for ACS use was not available. Therefore, we were unable to distinguish between clearly indicated and potentially precautionary administrations, which may have influenced our estimates. Finally, the generalizability of our findings to other populations or regions may be limited by the single-center nature of our data.

## Conclusion

ACS exposure, especially multiple courses or a last dose-to-delivery interval of 14 days or more, was associated with higher risks of metabolic, infectious/inflammatory, and neurological morbidities among infants born at 34 weeks of gestation or later. Additionally, the timing of the first dose of ACS, regardless of the specific gestational age at initiation, was associated with an increased risk of specific neonatal morbidities. No association was found between ACS exposure and neonatal morbidity in infants born before 34 weeks of gestation. These findings underscore the importance of carefully considering the timing and courses of ACS, and administering ACS at recommended gestational ages following thorough assessment of preterm birth risk, in order to avoid unnecessary exposure and minimize neonatal morbidity.

## Data Availability

The original contributions presented in the study are included in the article/Supplementary Material, further inquiries can be directed to the corresponding authors.
