# Supplementary material for: Associations between antenatal corticosteroids and neonatal morbidities in a prospective cohort: role of course, timing, and gestational age
Source: Front Pediatr. 2026 Mar 11;14:1784451. doi: 10.3389/fped.2026.1784451 (PMC13013305; doi:10.3389/fped.2026.1784451)
Supplement: Supplementary file 1 [file Supplementaryfile1.docx]

***Supplementary Material***

**Supplementary Table S1.** International Classification of Diseases, Tenth Revision (ICD-10) codes for neonatal and maternal morbidities.

**Supplementary Table S2.** Characteristics of the included and excluded population.

**Supplementary Table S3.** Characteristics of the study population according to numbers of ACS courses.

**Supplementary Table S4.** Characteristics of the study population according to gestational age at the first dose of ACS.

**Supplementary Table S5.** Characteristics of the study population according to last dose-to-delivery interval (days).

**Supplementary Table S6.** Association between numbers of ACS courses and neonatal morbidities stratified by gestational age at birth.

**Supplementary Table S7.** Association between gestational age at the first dose of ACS and neonatal morbidities stratified by gestational age at birth.

**Supplementary Table S8.** Association between last dose-to-delivery interval (days) and neonatal morbidities stratified by gestational age at birth.

| **Supplementary Table S1. International Classification of Diseases, Tenth Revision (ICD-10) codes for neonatal and maternal morbidities.** | |
| --- | --- |
| **Diagnoses** | **ICD-10** |
| **Neonatal morbidities** |  |
| **Asphyxia** | P21 |
| **Respiratory morbidity** |  |
| Respiratory distress syndrome | P22.0 |
| Respiratory failure | P28.5 |
| Meconium aspiration syndrome | P24.0 |
| Transient tachypnea | P22.1 |
| Others | P27.1 (Bronchopulmonary dysplasia originating in the perinatal period). P29.3 (Persistent fetal circulation, Pulmonary hypertension of newborn (persistent)) |
| **Metabolic morbidity** |  |
| Hypoglycemia | P70.4 |
| hyperbilirubinemia | P59.0, P59.9 |
| **Infectious/Inflammatory morbidity** |  |
| Sepsis | P36 |
| Necrotizing enterocolitis | P77 |
| Respiratory tract infection | P23, J00-J06, J20-J22 |
| Conjunctivitis | P39.1 |
| Others | L01 (Impetigo), L08 (Other local infections of skin and subcutaneous tissue), L44.4(Infantile papular acrodermatitis), P35-P39 (Infections specific to the perinatal period) |
| **Neurological morbidity** |  |
| Seizure | P90 |
| Intracranial hemorrhage | P52 |
| Hypoxic-ischemic encephalopathy | P91.6 |
| Periventricular leukomalacia | P91.2 |
|  |  |
| **Maternal morbidity** |  |
| Hypertensive diseases | I10-I15, O10, O11, O14, O15 |
| Diabetic diseases | E10-E14, O24 |

| **Supplementary Table S2. Characteristics of the included and excluded population.** | | | | |
| --- | --- | --- | --- | --- |
| **Characteristics** | **Overall (n [%])** | **Included population (n [%])** | **Excluded population (n [%])** | ***P* value^b^** |
| **Total ^a^** | 106,173 (100.00) | 78,642 (74.07) | 27,531 (25.93) |  |
| **Mothers** |  |  |  |  |
| **Age at delivery** |  |  |  |  |
| As continuous variable (mean [SD]) | 30.40 (4.11) | 30.38 (4.07) | 30.45 (4.20) | 0.009 |
| <25 | 6740 (6.35) | 4912 (6.25) | 1828 (6.64) | <0.001 |
| 25–29 | 39,520 (37.22) | 29,477 (37.48) | 10,043 (36.48) |  |
| 30–34 | 43,269 (40.75) | 32,101 (40.82) | 11,168 (40.57) |  |
| ≥35 | 16,644 (15.68) | 12,152 (15.45) | 4492 (16.32) |  |
| **Educational level** |  |  |  |  |
| Junior high school or below | 8905 (8.39) | 6028 (7.67) | 2877 (10.45) | <0.001 |
| Senior high school or vocational school | 15,644 (14.73) | 11,193 (14.23) | 4451 (16.17) |  |
| University or above | 74,966 (70.61) | 57,204 (72.74) | 17,762 (64.52) |  |
| Missing | 6658 (6.27) | 4217 (5.36) | 2441 (8.87) |  |
| **Parity** |  |  |  |  |
| 1 | 55,902 (52.65) | 42,535 (54.09) | 13,367 (48.55) | <0.001 |
| 2 | 42,173 (39.72) | 30,862 (39.24) | 11,311 (41.08) |  |
| ≥3 | 8087 (7.62) | 5241 (6.66) | 2846 (10.34) |  |
| Missing | 11 (0.01) | 4 (0.01) | 7 (0.03) |  |
| **Mode of conception** |  |  |  |  |
| Naturally conceived | 97,500 (91.83) | 72,819 (92.60) | 24,681 (89.65) | <0.001 |
| ART | 4198 (3.95) | 3031 (3.85) | 1167 (4.24) |  |
| Missing | 4475 (4.21) | 2792 (3.55) | 1683 (6.11) |  |
| **Pre-pregnancy BMI** |  |  |  |  |
| As continuous variable (median [IQR]) | 20.63 (19.07, 22.60) | 20.58 (19.05, 22.55) | 20.70 (19.13, 22.85) | <0.001 |
| <18.5 (underweight) | 17,349 (16.34) | 13,113 (16.67) | 4236 (15.39) | <0.001 |
| 18.5–23.9 (normal weight) | 69,339 (65.31) | 52,085 (66.23) | 17,254 (62.67) |  |
| 24.0–27.9 (overweight) | 12,093 (11.39) | 8660 (11.01) | 3433 (12.47) |  |
| ≥28.0 (obesity) | 2552 (2.40) | 1772 (2.25) | 780 (2.83) |  |
| Missing | 4840 (4.56) | 3012 (3.83) | 1828 (6.64) |  |
| **Diabetic disease** |  |  |  |  |
| No | 84,664 (79.74) | 62,903 (79.99) | 21,761 (79.04) | <0.001 |
| Yes | 21,509 (20.26) | 15,739 (20.01) | 5770 (20.96) |  |
| **Hypertensive disease** |  |  |  |  |
| No | 98,854 (93.11) | 73,864 (93.92) | 24,990 (90.77) | <0.001 |
| Yes | 7319 (6.89) | 4778 (6.08) | 2541 (9.23) |  |
| **Vaginal bleeding during early pregnancy** |  |  |  |  |
| No | 66,498 (62.63) | 50,482 (64.19) | 16,016 (58.17) | <0.001 |
| Yes | 15,094 (14.22) | 11,302 (14.37) | 3792 (13.77) |  |
| Missing | 24,581 (23.15) | 16,858 (21.44) | 7723 (28.05) |  |
| **Mode of delivery** |  |  |  |  |
| Vaginal delivery | 68,132 (64.17) | 51,436 (65.41) | 16,696 (60.64) | <0.001 |
| Elective cesarean section | 22,531 (21.22) | 15,737 (20.01) | 6794 (24.68) |  |
| Emergency cesarean section | 15,509 (14.61) | 11,469 (14.58) | 4040 (14.67) |  |
| Missing | 1 (0.00) | 0 (0.00) | 1 (0.00) |  |
| **Infants** |  |  |  |  |
| **Sex** |  |  |  |  |
| Male | 56,605 (53.31) | 41,927 (53.31) | 14,678 (53.31) | >0.999 |
| Female | 49,568 (46.69) | 36,715 (46.69) | 12,853 (46.69) |  |
| **Gestational age at birth (weeks)** |  |  |  |  |
| As continuous variable (median [IQR]) | 39.29 (38.57, 40.14) | 39.43 (38.71, 40.14) | 39.14 (38.29, 40.00) | 0.063 |
| <34 | 1367 (1.29) | 143 (0.18) | 1224 (4.45) | <0.001 |
| 34–36 | 4100 (3.86) | 2015 (2.56) | 2085 (7.57) |  |
| 37–38 | 30,661 (28.88) | 22,669 (28.83) | 7992 (29.03) |  |
| 39–40 | 62,612 (58.97) | 48,057 (61.11) | 14,555 (52.87) |  |
| ≥41 | 7432 (7.00) | 5758 (7.32) | 1674 (6.08) |  |
| Missing | 1 (0.00) | 0 (0.00) | 1 (0.00) |  |
| **Birth weight for gestational age (percentile)** |  |  |  |  |
| <10^th^ | 9080 (8.55) | 6303 (8.01) | 2777 (10.09) | <0.001 |
| 10^th^– 24^th^ | 16,256 (15.31) | 12,055 (15.33) | 4201 (15.26) |  |
| 25^th^– 74^th^ | 54,089 (50.94) | 40,452 (51.44) | 13,637 (49.53) |  |
| 75^th^– 89^th^ | 16,154 (15.21) | 12,026 (15.29) | 4128 (14.99) |  |
| ≥90^th^ | 10,593 (9.98) | 7806 (9.93) | 2787 (10.12) |  |
| Missing | 1 (0.00) | 0 (0.00) | 1 (0.00) |  |
| **Calendar year of birth** |  |  |  |  |
| 2018–2019 | 28,603 (26.94) | 21,976 (27.94) | 6627 (24.07) | <0.001 |
| 2020 | 17,712 (16.68) | 13,133 (16.70) | 4579 (16.63) |  |
| 2021 | 17,582 (16.56) | 13,554 (17.24) | 4028 (14.63) |  |
| 2022 | 17,236 (16.23) | 12,423 (15.80) | 4813 (17.48) |  |
| 2023–2024 | 25,040 (23.58) | 17,556 (22.32) | 7484 (27.18) |  |
| Abbreviations: ART=assisted reproductive technology; BMI=body mass index; IQR=interquartile range; SD=standard deviation. | | | | |
| ^a^ Number and row percentage. | | | | |
| ^b^ Continuous variables were compared using t test or Mann-Whitney U test; categorical variables were compared using chi-squared test. | | | | |

| **Supplementary Table S3. Characteristics of the study population according to numbers of ACS courses.** | | | | | |
| --- | --- | --- | --- | --- | --- |
| **Characteristics** | **Numbers of ACS courses (n[%])** | | | | P value^b^ |
|  | **Unexposed** | **Incomplete course** | **Single course** | **Multiple courses** |  |
| Total ^a^ | 75,815 (96.41) | 390 (0.50) | 2144 (2.73) | 293 (0.37) |  |
| **Mothers** |  |  |  |  |  |
| **Age at delivery** |  |  |  |  |  |
| As continuous variable (mean [SD]) | 30.36 (4.06) | 31.74 (4.69) | 30.59 (4.39) | 31.30 (4.45) | <0.001 |
| <25 | 4737 (6.25) | 15 (3.85) | 149 (6.95) | 11 (3.75) | <0.001 |
| 25–29 | 28,518 (37.62) | 107 (27.44) | 758 (35.35) | 94 (32.08) |  |
| 30–34 | 30,958 (40.83) | 176 (45.13) | 841 (39.23) | 126 (43.00) |  |
| ≥35 | 11,602 (15.30) | 92 (23.59) | 396 (18.47) | 62 (21.16) |  |
| **Educational level** |  |  |  |  |  |
| Junior high school or below | 5777 (7.62) | 41 (10.51) | 182 (8.49) | 28 (9.56) | <0.001 |
| Senior high school or vocational school | 10,802 (14.25) | 64 (16.41) | 293 (13.67) | 34 (11.60) |  |
| University or above | 55,231 (72.85) | 251 (64.36) | 1520 (70.90) | 202 (68.94) |  |
| Missing | 4005 (5.28) | 34 (8.72) | 149 (6.95) | 29 (9.90) |  |
| **Parity** |  |  |  |  |  |
| 1 | 40,928 (53.98) | 198 (50.77) | 1232 (57.46) | 177 (60.41) | <0.001 |
| 2 | 29,856 (39.38) | 148 (37.95) | 764 (35.63) | 94 (32.08) |  |
| ≥3 | 5027 (6.63) | 44 (11.28) | 148 (6.90) | 22 (7.51) |  |
| Missing | 4 (0.01) | 0 (0.00) | 0 (0.00) | 0 (0.00) |  |
| **Mode of conception** |  |  |  |  |  |
| Naturally conceived | 70,371 (92.82) | 340 (87.18) | 1876 (87.50) | 232 (79.18) | <0.001 |
| ART | 2811 (3.71) | 23 (5.90) | 159 (7.42) | 38 (12.97) |  |
| Missing | 2633 (3.47) | 27 (6.92) | 109 (5.08) | 23 (7.85) |  |
| **Pre-pregnancy BMI** |  |  |  |  |  |
| As continuous variable (median [IQR]) | 20.57 (19.07, 22.55) | 21.02 (19.20, 23.50) | 20.56 (18.87, 22.77) | 20.39 (18.98, 22.86) | 0.026 |
| <18.5 (underweight) | 12,598 (16.62) | 58 (14.87) | 403 (18.80) | 54 (18.43) | <0.001 |
| 18.5–23.9 (normal weight) | 50,380 (66.45) | 230 (58.97) | 1306 (60.91) | 169 (57.68) |  |
| 24.0–27.9 (overweight) | 8298 (10.95) | 56 (14.36) | 267 (12.45) | 39 (13.31) |  |
| ≥28.0 (obesity) | 1690 (2.23) | 18 (4.62) | 57 (2.66) | 7 (2.39) |  |
| Missing | 2849 (3.76) | 28 (7.18) | 111 (5.18) | 24 (8.19) |  |
| **Diabetic disease** |  |  |  |  |  |
| No | 60,825 (80.23) | 260 (66.67) | 1605 (74.86) | 213 (72.70) | <0.001 |
| Yes | 14,990 (19.77) | 130 (33.33) | 539 (25.14) | 80 (27.30) |  |
| **Hypertensive disease** |  |  |  |  |  |
| No | 71,357 (94.12) | 350 (89.74) | 1903 (88.76) | 254 (86.69) | <0.001 |
| Yes | 4458 (5.88) | 40 (10.26) | 241 (11.24) | 39 (13.31) |  |
| **Vaginal bleeding during early pregnancy** |  |  |  |  |  |
| No | 48,901 (64.50) | 205 (52.56) | 1225 (57.14) | 151 (51.54) | <0.001 |
| Yes | 10,719 (14.14) | 77 (19.74) | 432 (20.15) | 74 (25.26) |  |
| Missing | 16,195 (21.36) | 108 (27.69) | 487 (22.71) | 68 (23.21) |  |
| **Mode of delivery** |  |  |  |  |  |
| Vaginal delivery | 49,941 (65.87) | 204 (52.31) | 1156 (53.92) | 135 (46.08) | <0.001 |
| Elective cesarean section | 14,953 (19.72) | 141 (36.15) | 560 (26.12) | 83 (28.33) |  |
| Emergency cesarean section | 10,921 (14.40) | 45 (11.54) | 428 (19.96) | 75 (25.60) |  |
| **Infants** |  |  |  |  |  |
| **Sex** |  |  |  |  |  |
| Male | 40,380 (53.26) | 225 (57.69) | 1157 (53.96) | 165 (56.31) | 0.211 |
| Female | 35,435 (46.74) | 165 (42.31) | 987 (46.04) | 128 (43.69) |  |
| **Gestational age at birth (weeks)** |  |  |  |  |  |
| As continuous variable (median [IQR]) | 39.43 (38.71, 40.14) | 36.43 (35.29, 38.14) | 38.57 (37.14, 39.43) | 37.00 (35.71, 38.43) | <0.001 |
| <34 | 46 (0.06) | 26 (6.67) | 53 (2.47) | 18 (6.14) | <0.001 |
| 34–36 | 1329 (1.75) | 215 (55.13) | 349 (16.28) | 122 (41.64) |  |
| 37–38 | 21,579 (28.46) | 87 (22.31) | 906 (42.26) | 97 (33.11) |  |
| 39–40 | 47,160 (62.20) | 60 (15.38) | 782 (36.47) | 55 (18.77) |  |
| ≥41 | 5701 (7.52) | 2 (0.51) | 54 (2.52) | 1 (0.34) |  |
| **Birth weight for gestational age (percentile)** |  |  |  |  |  |
| <10^th^ | 6073 (8.01) | 25 (6.41) | 186 (8.68) | 19 (6.48) | 0.137 |
| 10^th^–24^th^ | 11,664 (15.38) | 47 (12.05) | 309 (14.41) | 35 (11.95) |  |
| 25^th^–74^th^ | 38,979 (51.41) | 199 (51.03) | 1121 (52.29) | 153 (52.22) |  |
| 75^th^–89^th^ | 11,579 (15.27) | 70 (17.95) | 327 (15.25) | 50 (17.06) |  |
| ≥90^th^ | 7520 (9.92) | 49 (12.56) | 201 (9.38) | 36 (12.29) |  |
| **Calendar year of birth** |  |  |  |  |  |
| 2018–2019 | 21,289 (28.08) | 106 (27.18) | 507 (23.65) | 74 (25.26) | <0.001 |
| 2020 | 12,762 (16.83) | 51 (13.08) | 287 (13.39) | 33 (11.26) |  |
| 2021 | 13,025 (17.18) | 77 (19.74) | 398 (18.56) | 54 (18.43) |  |
| 2022 | 11,903 (15.70) | 76 (19.49) | 389 (18.14) | 55 (18.77) |  |
| 2023–2024 | 16,836 (22.21) | 80 (20.51) | 563 (26.26) | 77 (26.28) |  |
| Abbreviations: ART=assisted reproductive technology; BMI=body mass index; IQR=interquartile range; SD=standard deviation. | | | | | |
| ^a^ Number and row percentage. | | | | | |
| ^b^ Continuous variables were compared using ANOVA or Kruskal-Wallis test; categorical variables were compared using chi-squared test or Fisher’s exact test. | | | | | |

| **Supplementary Table S4. Characteristics of the study population according to gestational age at the first dose of ACS.** | | | | | |
| --- | --- | --- | --- | --- | --- |
| **Characteristics** | **Gestational age at the first dose (weeks) [n(%)]** | | | | ***P* value^b^** |
|  | **Unexposed** | **<34** | **34–36** | **37**–**39** |  |
| **Total ^a^** | 75,815 (96.41) | 1744 (2.22) | 1051 (1.34) | 32 (0.04) |  |
| **Mothers** |  |  |  |  |  |
| **Age at delivery** |  |  |  |  |  |
| As continuous variable (mean [SD]) | 30.36 (4.06) | 30.56 (4.41) | 31.22 (4.49) | 32.19 (4.75) | <0.001 |
| <25 | 4737 (6.25) | 121 (6.94) | 52 (4.95) | 2 (6.25) | <0.001 |
| 25–29 | 28,518 (37.62) | 615 (35.26) | 339 (32.25) | 5 (15.63) |  |
| 30–34 | 30,958 (40.83) | 702 (40.25) | 425 (40.44) | 16 (50.00) |  |
| ≥35 | 11,602 (15.30) | 306 (17.55) | 235 (22.36) | 9 (28.13) |  |
| **Educational level** |  |  |  |  |  |
| Junior high school or below | 5777 (7.62) | 141 (8.08) | 106 (10.09) | 4 (12.50) | <0.001 |
| Senior high school or vocational school | 10,802 (14.25) | 225 (12.90) | 163 (15.51) | 3 (9.38) |  |
| University or above | 55,231 (72.85) | 1242 (71.22) | 709 (67.46) | 22 (68.75) |  |
| Missing | 4005 (5.28) | 136 (7.80) | 73 (6.95) | 3 (9.38) |  |
| **Parity** |  |  |  |  |  |
| 1 | 40,928 (53.98) | 1056 (60.55) | 534 (50.81) | 17 (53.13) | <0.001 |
| 2 | 29,856 (39.38) | 595 (34.12) | 401 (38.15) | 10 (31.25) |  |
| ≥3 | 5027 (6.63) | 93 (5.33) | 116 (11.04) | 5 (15.63) |  |
| Missing | 4 (0.01) | 0 (0.00) | 0 (0.00) | 0 (0.00) |  |
| **Mode of conception** |  |  |  |  |  |
| Naturally conceived | 70,371 (92.82) | 1499 (85.95) | 921 (87.63) | 28 (87.50) | <0.001 |
| ART | 2811 (3.71) | 139 (7.97) | 80 (7.61) | 1 (3.13) |  |
| Missing | 2633 (3.47) | 106 (6.08) | 50 (4.76) | 3 (9.38) |  |
| **Pre-pregnancy BMI** |  |  |  |  |  |
| As continuous variable (median [IQR]) | 20.57 (19.07, 22.55) | 20.42 (18.80, 22.43) | 20.83 (19.14, 23.23) | 22.58 (19.76, 25.11) | <0.001 |
| <18.5 (underweight) | 12,598 (16.62) | 327 (18.75) | 184 (17.51) | 4 (12.50) | <0.001 |
| 18.5–23.9 (normal weight) | 50,380 (66.45) | 1066 (61.12) | 626 (59.56) | 13 (40.63) |  |
| 24.0–27.9 (overweight) | 8298 (10.95) | 200 (11.47) | 151 (14.37) | 11 (34.38) |  |
| ≥28.0 (obesity ) | 1690 (2.23) | 42 (2.41) | 39 (3.71) | 1 (3.13) |  |
| Missing | 2849 (3.76) | 109 (6.25) | 51 (4.85) | 3 (9.38) |  |
| **Diabetic disease** |  |  |  |  |  |
| No | 60,825 (80.23) | 1283 (73.57) | 770 (73.26) | 25 (78.13) | <0.001 |
| Yes | 14,990 (19.77) | 461 (26.43) | 281 (26.74) | 7 (21.88) |  |
| **Hypertensive disease** |  |  |  |  |  |
| No | 71,357 (94.12) | 1588 (91.06) | 891 (84.78) | 28 (87.50) | <0.001 |
| Yes | 4458 (5.88) | 156 (8.94) | 160 (15.22) | 4 (12.50) |  |
| **Vaginal bleeding during early pregnancy** |  |  |  |  |  |
| No | 48,901 (64.50) | 955 (54.76) | 608 (57.85) | 18 (56.25) | <0.001 |
| Yes | 10,719 (14.14) | 384 (22.02) | 196 (18.65) | 3 (9.38) |  |
| Missing | 16,195 (21.36) | 405 (23.22) | 247 (23.50) | 11 (34.38) |  |
| **Mode of delivery** |  |  |  |  |  |
| Vaginal delivery | 49,941 (65.87) | 1013 (58.08) | 477 (45.39) | 5 (15.63) | <0.001 |
| Elective cesarean section | 14,953 (19.72) | 450 (25.80) | 321 (30.54) | 13 (40.63) |  |
| Emergency cesarean section | 10,921 (14.40) | 281 (16.11) | 253 (24.07) | 14 (43.75) |  |
| **Infants** |  |  |  |  |  |
| **Sex** |  |  |  |  |  |
| Male | 40,380 (53.26) | 945 (54.19) | 591 (56.23) | 11 (34.38) | 0.032 |
| Female | 35,435 (46.74) | 799 (45.81) | 460 (43.77) | 21 (65.63) |  |
| **Gestational age at birth (weeks)** |  |  |  |  |  |
| As continuous variable (median [IQR]) | 39.43 (38.71, 40.14) | 38.57 (37.29, 39.57) | 37.14 (36.29, 38.71) | 37.71 (37.43, 38.22) | <0.001 |
| <34 | 46 (0.06) | 97 (5.56) | 0 (0.00) | 0 (0.00) | <0.001 |
| 34–36 | 1329 (1.75) | 261 (14.97) | 425 (40.44) | 0 (0.00) |  |
| 37–38 | 21,579 (28.46) | 659 (37.79) | 404 (38.44) | 27 (84.38) |  |
| 39–40 | 47,160 (62.20) | 679 (38.93) | 213 (20.27) | 5 (15.63) |  |
| ≥41 | 5701 (7.52) | 48 (2.75) | 9 (0.86) | 0 (0.00) |  |
| **Birth weight for gestational age (percentile)** |  |  |  |  |  |
| <10^th^ | 6073 (8.01) | 137 (7.86) | 84 (7.99) | 9 (28.13) | 0.003 |
| 10^th^– 24^th^ | 11,664 (15.38) | 229 (13.13) | 157 (14.94) | 5 (15.63) |  |
| 25^th^– 74^th^ | 38,979 (51.41) | 937 (53.73) | 527 (50.14) | 9 (28.13) |  |
| 75^th^– 89^th^ | 11,579 (15.27) | 276 (15.83) | 166 (15.79) | 5 (15.63) |  |
| ≥90^th^ | 7520 (9.92) | 165 (9.46) | 117 (11.13) | 4 (12.50) |  |
| **Calendar year of birth** |  |  |  |  |  |
| 2018–2019 | 21,289 (28.08) | 417 (23.91) | 257 (24.45) | 13 (40.63) | <0.001 |
| 2020 | 12,762 (16.83) | 265 (15.19) | 103 (9.80) | 3 (9.38) |  |
| 2021 | 13,025 (17.18) | 310 (17.78) | 214 (20.36) | 5 (15.63) |  |
| 2022 | 11,903 (15.70) | 281 (16.11) | 233 (22.17) | 6 (18.75) |  |
| 2023–2024 | 16,836 (22.21) | 471 (27.01) | 244 (23.22) | 5 (15.63) |  |
| Abbreviations: ART=assisted reproductive technology; BMI=body mass index; IQR=interquartile range; SD=standard deviation. | | | | | |
| ^a^ Number and row percentage. | | | | | |
| ^b^ Continuous variables were compared using ANOVA or Kruskal-Wallis test; categorical variables were compared using chi-squared test or Fisher’s exact test. | | | | | |

| **Supplementary Table S5. Characteristics of the study population according to last dose-to-delivery interval (days).** | | | | | | | | | | | | | | |  |
| --- | --- | --- | --- | --- | --- | --- | --- | --- | --- | --- | --- | --- | --- | --- | --- |
| **Characteristics** | | | **Last dose-to-delivery interval (days) [n(%)]** | | | | | | | | | | | ***P* value^b^** |  |
|  |  |  | **Unexposed** | | **<2** | | **2**–**7** | | **8**–**13** | | | **≥14** | |  |  |
| **Total ^a^** | | | 75,815 (96.41) | | 251 (0.32) | | 333 (0.42) | | 149 (0.19) | | | 2094 (2.66) | |  |  |
| **Mothers** | | |  | |  | |  | |  | | |  | |  |  |
| **Age at delivery** | | |  | |  | |  | |  | | |  | |  |  |
| As continuous variable (mean [SD]) | | | 30.36 (4.06) | | 31.63 (4.53) | | 31.24 (5.06) | | 31.44 (4.79) | | | 30.61 (4.30) | | <0.001 |  |
| <25 | | | 4737 (6.25) | | 9 (3.59) | | 26 (7.81) | | 10 (6.71) | | | 130 (6.21) | | <0.001 |  |
| 25–29 | | | 28,518 (37.62) | | 70 (27.89) | | 106 (31.83) | | 41 (27.52) | | | 742 (35.43) | |  |  |
| 30–34 | | | 30,958 (40.83) | | 119 (47.41) | | 114 (34.23) | | 57 (38.26) | | | 853 (40.74) | |  |  |
| ≥35 | | | 11,602 (15.30) | | 53 (21.12) | | 87 (26.13) | | 41 (27.52) | | | 369 (17.62) | |  |  |
| **Educational level** | | |  | |  | |  | |  | | |  | |  |  |
| Junior high school or below | | | 5777 (7.62) | | 28 (11.16) | | 33 (9.91) | | 17 (11.41) | | | 173 (8.26) | | <0.001 |  |
| Senior high school or vocational school | | | 10,802 (14.25) | | 48 (19.12) | | 57 (17.12) | | 14 (9.40) | | | 272 (12.99) | |  |  |
| University or above | | | 55,231 (72.85) | | 154 (61.35) | | 212 (63.66) | | 109 (73.15) | | | 1498 (71.54) | |  |  |
| Missing | | | 4005 (5.28) | | 21 (8.37) | | 31 (9.31) | | 9 (6.04) | | | 151 (7.21) | |  |  |
| **Parity** | | |  | |  | |  | |  | | |  | |  |  |
| 1 | | | 40,928 (53.98) | | 134 (53.39) | | 185 (55.56) | | 71 (47.65) | | | 1217 (58.12) | | <0.001 |  |
| 2 | | | 29,856 (39.38) | | 88 (35.06) | | 110 (33.03) | | 60 (40.27) | | | 748 (35.72) | |  |  |
| ≥3 | | | 5027 (6.63) | | 29 (11.55) | | 38 (11.41) | | 18 (12.08) | | | 129 (6.16) | |  |  |
| Missing | | | 4 (0.01) | | 0 (0.00) | | 0 (0.00) | | 0 (0.00) | | | 0 (0.00) | |  |  |
| **Mode of conception** | | |  | |  | |  | |  | | |  | |  |  |
| Naturally conceived | | | 70,371 (92.82) | | 218 (86.85) | | 283 (84.98) | | 130 (87.25) | | | 1817 (86.77) | | <0.001 |  |
| ART | | | 2811 (3.71) | | 15 (5.98) | | 30 (9.01) | | 13 (8.72) | | | 162 (7.74) | |  |  |
| Missing | | | 2633 (3.47) | | 18 (7.17) | | 20 (6.01) | | 6 (4.03) | | | 115 (5.49) | |  |  |
| **Pre-pregnancy BMI** | | |  | |  | |  | |  | | |  | |  |  |
| As continuous variable (median [IQR]) | | | 20.57 (19.07, 22.55) | | 21.09 (19.23, 23.44) | | 21.21 (19.49, 23.85) | | 21.39 (18.84, 23.44) | | | 20.43 (18.82, 22.44) | | <0.001 |  |
| <18.5 (underweight) | | | 12,598 (16.62) | | 34 (13.55) | | 49 (14.71) | | 28 (18.79) | | | 404 (19.29) | | <0.001 |  |
| 18.5–23.9 (normal weight) | | | 50,380 (66.45) | | 155 (61.75) | | 185 (55.56) | | 85 (57.05) | | | 1280 (61.13) | |  |  |
| 24.0–27.9 (overweight) | | | 8298 (10.95) | | 35 (13.94) | | 66 (19.82) | | 25 (16.78) | | | 236 (11.27) | |  |  |
| ≥28.0 (obesity) | | | 1690 (2.23) | | 9 (3.59) | | 12 (3.60) | | 6 (4.03) | | | 55 (2.63) | |  |  |
| Missing | | | 2849 (3.76) | | 18 (7.17) | | 21 (6.31) | | 5 (3.36) | | | 119 (5.68) | |  |  |
| **Diabetic disease** | | |  | |  | |  | |  | | |  | |  |  |
| No | | | 60,825 (80.23) | | 168 (66.93) | | 247 (74.17) | | 106 (71.14) | | | 1557 (74.36) | | <0.001 |  |
| Yes | | | 14,990 (19.77) | | 83 (33.07) | | 86 (25.83) | | 43 (28.86) | | | 537 (25.64) | |  |  |
| **Hypertensive disease** | | |  | |  | |  | |  | | |  | |  |  |
| No | | | 71,357 (94.12) | | 218 (86.85) | | 262 (78.68) | | 113 (75.84) | | | 1,914 (91.40) | | <0.001 |  |
| Yes | | | 4458 (5.88) | | 33 (13.15) | | 71 (21.32) | | 36 (24.16) | | | 180 (8.60) | |  |  |
| **Vaginal bleeding during early pregnancy** | | |  | |  | |  | |  | | |  | |  |  |
| No | | | 48,901 (64.50) | | 131 (52.19) | | 179 (53.75) | | 80 (53.69) | | | 1191 (56.88) | | <0.001 |  |
| Yes | | | 10,719 (14.14) | | 46 (18.33) | | 50 (15.02) | | 36 (24.16) | | | 451 (21.54) | |  |  |
| Missing | | | 16,195 (21.36) | | 74 (29.48) | | 104 (31.23) | | 33 (22.15) | | | 452 (21.59) | |  |  |
| **Mode of delivery** | | |  | |  | |  | |  | | |  | |  |  |
| Vaginal delivery | | | 49,941 (65.87) | | 132 (52.59) | | 96 (28.83) | | 47 (31.54) | | | 1220 (58.26) | | <0.001 |  |
| Elective cesarean section | | | 14,953 (19.72) | | 101 (40.24) | | 132 (39.64) | | 49 (32.89) | | | 502 (23.97) | |  |  |
| Emergency cesarean section | | | 10,921 (14.40) | | 18 (7.17) | | 105 (31.53) | | 53 (35.57) | | | 372 (17.77) | |  |  |
| **Infants** | | |  | |  | |  | |  | | |  | |  |  |
| **Sex** | | |  | |  | |  | |  | | |  | |  |  |
| Male | | | 40,380 (53.26) | | 151 (60.16) | | 193 (57.96) | | 84 (56.38) | | | 1119 (53.44) | | 0.082 |  |
| Female | | | 35,435 (46.74) | | 100 (39.84) | | 140 (42.04) | | 65 (43.62) | | | 975 (46.56) | |  |  |
| **Gestational age at birth (weeks)** | | |  | |  | |  | |  | | |  | |  |  |
| As continuous variable (median [IQR]) | | | 39.43 (38.71, 40.14) | | 35.86 (34.86, 36.57) | | 36.00 (34.57, 36.86) | | 37.00 (35.86, 37.57) | | | 38.71 (37.71, 39.57) | | <0.001 |  |
| <34 | | | 46 (0.06) | | 26 (10.36) | | 49 (14.71) | | 6 (4.03) | | | 16 (0.76) | | <0.001 |  |
| 34–36 | | | 1329 (1.75) | | 203 (80.88) | | 206 (61.86) | | 61 (40.94) | | | 216 (10.32) | |  |  |
| 37–38 | | | 21,579 (28.46) | | 22 (8.76) | | 75 (22.52) | | 81 (54.36) | | | 912 (43.55) | |  |  |
| 39–40 | | | 47,160 (62.20) | | 0 (0.00) | | 3 (0.90) | | 1 (0.67) | | | 893 (42.65) | |  |  |
| ≥41 | | | 5701 (7.52) | | 0 (0.00) | | 0 (0.00) | | 0 (0.00) | | | 57 (2.72) | |  |  |
| **Birth weight for gestational age (percentile)** | | |  | |  | |  | |  | | |  | |  |  |
| <10^th^ | | | 6073 (8.01) | | 15 (5.98) | | 25 (7.51) | | 7 (4.70) | | | 183 (8.74) | | 0.113 |  |
| 10^th^–24^th^ | | | 11,664 (15.38) | | 23 (9.16) | | 42 (12.61) | | 29 (19.46) | | | 297 (14.18) | |  |  |
| 25^th^–74^th^ | | | 38,979 (51.41) | | 136 (54.18) | | 176 (52.85) | | 71 (47.65) | | | 1090 (52.05) | |  |  |
| 75^th^–89^th^ | | | 11,579 (15.27) | | 46 (18.33) | | 59 (17.72) | | 23 (15.44) | | | 319 (15.23) | |  |  |
| ≥90^th^ | | | 7520 (9.92) | | 31 (12.35) | | 31 (9.31) | | 19 (12.75) | | | 205 (9.79) | |  |  |
| **Calendar year of birth** | | |  | |  | |  | |  | | |  | |  |  |
| 2018–2019 | | | 21,289 (28.08) | | 76 (30.28) | | 104 (31.23) | | 32 (21.48) | | | 475 (22.68) | | <0.001 |  |
| 2020 | | | 12,762 (16.83) | | 26 (10.36) | | 42 (12.61) | | 15 (10.07) | | | 288 (13.75) | |  |  |
| 2021 | | | 13,025 (17.18) | | 49 (19.52) | | 55 (16.52) | | 35 (23.49) | | | 390 (18.62) | |  |  |
| 2022 | | | 11,903 (15.70) | | 51 (20.32) | | 63 (18.92) | | 31 (20.81) | | | 375 (17.91) | |  |  |
| 2023–2024 | | | 16,836 (22.21) | | 49 (19.52) | | 69 (20.72) | | 36 (24.16) | | | 566 (27.03) | |  |  |
| Abbreviations: ART=assisted reproductive technology; BMI=body mass index; IQR=interquartile range; SD=standard deviation. | | | | | | | | | | | | | | |  |
| ^a^ Number and row percentage. | | | | | | | | | | | | | | |  |
| ^b^ Continuous variables were compared using ANOVA or Kruskal-Wallis test; categorical variables were compared using chi-squared test or Fisher’s exact test. | | | | | | | | | | | | | | |  |
| **Supplementary Table S6. Association between numbers of ACS courses and neonatal morbidities stratified by gestational age at birth.** | | | | | | | | | | | | | | | |
| **Numbers of ACS courses** | **Gestational age at birth (weeks)** | | | | | | | | | | | | | | |
|  | **<34** | | | **34–36** | | | | **37–38** | | | **≥39** | | | | |
|  | **N of cases (rate)^a^** | **Adjusted OR (95 CI)^b^** | | **N of cases (rate)^a^** | | **Adjusted OR (95 CI)^b^** | | **N of cases (rate)^a^** | | **Adjusted OR (95 CI)^b^** | **N of cases (rate)^a^** | | **Adjusted OR (95 CI)^b^** | | |
| **Any morbidity** |  |  | |  | |  | |  | |  |  | |  | | |
| Unexposed | 38 (82.61) | Ref. | | 951 (71.56) | | Ref. | | 11739 (54.4) | | Ref. | 25931 (49.06) | | Ref. | | |
| Incomplete course | 25 (96.15) | 3.73 (0.29–47.96) | | 154 (71.63) | | 1.02 (0.73–1.41) | | 56 (64.37) | | 1.67 (1.08–2.60) | 36 (58.06) | | 1.45 (0.87–2.42) | | |
| Single course | 42 (79.25) | 0.81 (0.17–3.94) | | 241 (69.05) | | 1.02 (0.77–1.36) | | 531 (58.61) | | 1.26 (1.10–1.45) | 464 (55.5) | | 1.29 (1.12–1.49) | | |
| Multiple courses | 16 (88.89) | 1.02 (0.07–15.83) | | 95 (77.87) | | 1.59 (0.99–2.55) | | 61 (62.89) | | 1.46 (0.96–2.23) | 35 (62.5) | | 1.76 (1.02–3.03) | | |
| **Asphyxia** |  |  | |  | |  | |  | |  |  | |  | | |
| Unexposed | 4 (8.7) | Ref. | | 16 (1.2) | | Ref. | | 69 (0.32) | | Ref. | 254 (0.48) | | Ref. | | |
| Incomplete course | 2 (7.69) | NA | | 0 (0) | | NA | | 1 (1.15) | | 4.13 (0.58–29.65) | 0 (0) | | NA | | |
| Single course | 1 (1.89) | NA | | 2 (0.57) | | NA | | 3 (0.33) | | 1.16 (0.36–3.70) | 8 (0.96) | | 2.06 (1.01–4.19) | | |
| Multiple courses | 0 (0) | NA | | 3 (2.46) | | NA | | 0 (0) | | NA | 0 (0) | | NA | | |
| **Respiratory morbidity** |  |  | |  | |  | |  | |  |  | |  | | |
| Unexposed | 16 (34.78) | Ref. | | 39 (2.93) | | Ref. | | 57 (0.26) | | Ref. | 163 (0.31) | | Ref. | | |
| Incomplete course | 11 (42.31) | 1.63 (0.43–6.18) | | 15 (6.98) | | 2.16 (1.15–4.06) | | 0 (0) | | NA | 0 (0) | | NA | | |
| Single course | 17 (32.08) | 0.75 (0.22–2.50) | | 23 (6.59) | | 1.75 (0.98–3.13) | | 6 (0.66) | | 2.21 (0.98–4.98) | 2 (0.24) | | 0.74 (0.18–3.00) | | |
| Multiple courses | 6 (33.33) | 0.53 (0.08–3.51) | | 8 (6.56) | | 1.85 (0.82–4.16) | | 0 (0) | | NA | 0 (0) | | NA | | |
| **Metabolic morbidity** |  |  | |  | |  | |  | |  |  | |  | | |
| Unexposed | 25 (54.35) | Ref. | | 862 (64.86) | | Ref. | | 10518 (48.74) | | Ref. | 22035 (41.68) | | Ref. | | |
| Incomplete course | 18 (69.23) | 1.30 (0.41–4.10) | | 131 (60.93) | | 0.85 (0.62–1.16) | | 53 (60.92) | | 1.85 (1.20–2.85) | 32 (51.61) | | 1.50 (0.91–2.47) | | |
| Single course | 30 (56.6) | 0.79 (0.27–2.30) | | 205 (58.74) | | 0.91 (0.70–1.19) | | 473 (52.21) | | 1.23 (1.07–1.41) | 391 (46.77) | | 1.23 (1.06–1.41) | | |
| Multiple courses | 14 (77.78) | 3.08 (0.59–16.13) | | 79 (64.75) | | 1.09 (0.72–1.67) | | 56 (57.73) | | 1.49 (0.98–2.26) | 29 (51.79) | | 1.53 (0.91–2.57) | | |
| **Infectious/Inflammatory diseases** |  |  | |  | |  | |  | |  |  | |  | | |
| Unexposed | 21 (45.65) | Ref. | | 291 (21.9) | | Ref. | | 3674 (17.03) | | Ref. | 8945 (16.92) | | Ref. | | |
| Incomplete course | 14 (53.85) | 2.21 (0.59–8.25) | | 54 (25.12) | | 1.23 (0.87–1.74) | | 15 (17.24) | | 1.06 (0.60–1.85) | 11 (17.74) | | 1.07 (0.56–2.04) | | |
| Single course | 22 (41.51) | 1.13 (0.40–3.16) | | 88 (25.21) | | 1.26 (0.94–1.69) | | 185 (20.42) | | 1.28 (1.08–1.51) | 160 (19.14) | | 1.15 (0.96–1.36) | | |
| Multiple courses | 12 (66.67) | 3.80 (0.86–16.68) | | 35 (28.69) | | 1.53 (0.99–2.38) | | 22 (22.68) | | 1.50 (0.93–2.42) | 10 (17.86) | | 1.09 (0.55–2.16) | | |
| **Neurological morbidity** |  |  | |  | |  | |  | |  |  | |  | | |
| Unexposed | 3 (6.52) | Ref. | | 50 (3.76) | | Ref. | | 139 (0.64) | | Ref. | 258 (0.49) | | Ref. | | |
| Incomplete course | 2 (7.69) | NA | | 9 (4.19) | | 0.91 (0.42–1.96) | | 2 (2.3) | | 3.45 (0.81–14.73) | 2 (3.23) | | 6.86 (1.72–27.36) | | |
| Single course | 2 (3.77) | NA | | 20 (5.73) | | 1.38 (0.78–2.44) | | 8 (0.88) | | 1.18 (0.56–2.48) | 3 (0.36) | | 0.66 (0.21–2.05) | | |
| Multiple courses | 2 (11.11) | NA | | 11 (9.02) | | 2.01 (1.02–3.95) | | 3 (3.09) | | 5.65 (1.64–19.49) | 2 (3.57) | | 5.83 (1.34–25.27) | | |
| Abbreviations: OR=Odds ratio; CI=confidence interval; NA=Not available due to convergence was not achieved due to the limited number of cases. | | | | | | | | | | | | | | | |
| ^a^ Rate represents the number of cases per thousand infants. | | | | | | | | | | | | | | | |
| ^b^ Model adjusted for maternal age at delivery, educational level, parity, mode of conception, pre-pregnancy BMI, hypertensive diseases, diabetic diseases, mode of delivery and vaginal bleeding during early pregnancy of the mother, as well as sex, birth weight for gestational age (percentile), and calendar year of birth of the infant, after multiple imputation by chained equations. | | | | | | | | | | | | | | | |

| **Supplementary Table S7. Association between gestational age at the first dose of ACS and neonatal morbidities stratified by gestational age at birth.** | | | | | | | | |
| --- | --- | --- | --- | --- | --- | --- | --- | --- |
| **Gestational age at the first dose (weeks)** | **Gestational age at birth (weeks)** | | | | | | | |
|  | **<34** | | **34–36** | | **37–38** | | **≥39** | |
|  | **N of cases (rate)^a^** | **Adjusted OR (95 CI)^b^** | **N of cases (rate)^a^** | **Adjusted OR (95 CI)^b^** | **N of cases (rate)^a^** | **Adjusted OR (95 CI)^b^** | **N of cases (rate)^a^** | **Adjusted OR (95 CI)^b^** |
| **Any morbidity** |  |  |  |  |  |  |  |  |
| Unexposed | 38 (82.61) | Ref. | 951 (71.56) | Ref. | 11739 (54.4) | Ref. | 25931 (49.06) | Ref. |
| <34 | 83 (85.57) | 1.15 (0.25–5.28) | 193 (73.95) | 1.22 (0.88–1.69) | 393 (59.64) | 1.27 (1.08–1.50) | 413 (56.81) | 1.35 (1.16–1.57) |
| 34–36 | - | - | 297 (69.88) | 1.02 (0.78–1.32) | 237 (58.66) | 1.33 (1.08–1.63) | 120 (54.05) | 1.30 (0.99–1.69) |
| 37–39 | - | - | - | - | 18 (66.67) | 2.11 (0.95–4.65) | 2 (40) | 0.62 (0.11–3.35) |
| **Asphyxia** |  |  |  |  |  |  |  |  |
| Unexposed | 4 (8.7) | Ref. | 16 (1.2) | Ref. | 69 (0.32) | Ref. | 254 (0.48) | Ref. |
| <34 | 3 (3.09) | NA | 4 (1.53) | NA | 3 (0.46) | 1.54 (0.48–4.92) | 6 (0.83) | 1.69 (0.75–3.80) |
| 34–36 | - | - | 1 (0.24) | NA | 0 (0) | NA | 2 (0.9) | 2.24 (0.54–9.24) |
| 37–39 | - | - | - | - | 1 (3.7) | 11.04 (1.47–83.00) | 0 (0) | NA |
| **Respiratory morbidity** |  |  |  |  |  |  |  |  |
| Unexposed | 16 (34.78) | Ref. | 39 (2.93) | Ref. | 57 (0.26) | Ref. | 163 (0.31) | Ref. |
| <34 | 34 (35.05) | 0.87 (0.34–2.25) | 19 (7.28) | 2.19 (1.22–3.94) | 1 (0.15) | 0.51 (0.07–3.67) | 2 (0.28) | 0.82 (0.20–3.34) |
| 34–36 | - | - | 27 (6.35) | 1.74 (1.00–3.02) | 4 (0.99) | 3.39 (1.21–9.52) | 0 (0) | NA |
| 37–39 | - | - | - | - | 1 (3.7) | 10.91 (2.06–57.68) | 0 (0) | NA |
| **Metabolic morbidity** |  |  |  |  |  |  |  |  |
| Unexposed | 25 (54.35) | Ref. | 862 (64.86) | Ref. | 10518 (48.74) | Ref. | 22035 (41.68) | Ref. |
| <34 | 62 (63.92) | 1.21 (0.53–2.79) | 162 (62.07) | 0.96 (0.71–1.28) | 351 (53.26) | 1.24 (1.06–1.46) | 344 (47.32) | 1.23 (1.06–1.44) |
| 34–36 | - | - | 253 (59.53) | 0.89 (0.70–1.14) | 213 (52.72) | 1.31 (1.07–1.61) | 106 (47.75) | 1.35 (1.04–1.77) |
| 37–39 | - | - | - | - | 18 (66.67) | 2.73 (1.23–6.05) | 2 (40) | 0.82 (0.15–4.50) |
| **Infectious/Inflammatory diseases** |  |  |  |  |  |  |  |  |
| Unexposed | 21 (45.65) | Ref. | 291 (21.9) | Ref. | 3674 (17.03) | Ref. | 8945 (16.92) | Ref. |
| <34 | 48 (49.48) | 1.73 (0.71–4.23) | 74 (28.35) | 1.43 (1.04–1.97) | 135 (20.49) | 1.28 (1.05–1.56) | 141 (19.39) | 1.16 (0.96–1.40) |
| 34–36 | - | - | 103 (24.24) | 1.20 (0.92–1.58) | 84 (20.79) | 1.32 (1.03–1.68) | 40 (18.02) | 1.09 (0.78–1.54) |
| 37–39 | - | - | - | - | 3 (11.11) | 0.72 (0.22–2.36) | 0 (0) | NA |
| **Neurological morbidity** |  |  |  |  |  |  |  |  |
| Unexposed | 3 (6.52) | Ref. | 50 (3.76) | Ref. | 139 (0.64) | Ref. | 258 (0.49) | Ref. |
| <34 | 6 (6.19) | NA | 24 (9.2) | 2.27 (1.37–3.77) | 9 (1.37) | 1.84 (0.92–3.71) | 6 (0.83) | 1.50 (0.67–3.38) |
| 34–36 | - | - | 16 (3.76) | 0.80 (0.42–1.51) | 3 (0.74) | 1.08 (0.33–3.50) | 1 (0.45) | 0.83 (0.11–6.02) |
| 37–39 | - | - | - | - | 1 (3.7) | 4.66 (0.54–40.27) | 0 (0) | NA |
| Abbreviations: OR=Odds ratio; CI=confidence interval; NA=Not available due to convergence was not achieved due to the limited number of cases. | | | | | | | | |
| ^a^ Rate represents the number of cases per thousand infants. | | | | | | | | |
| ^b^ Model adjusted for maternal age at delivery, educational level, parity, mode of conception, pre-pregnancy BMI, hypertensive diseases, diabetic diseases, mode of delivery and vaginal bleeding during early pregnancy of the mother, as well as sex, birth weight for gestational age (percentile), and calendar year of birth of the infant, after multiple imputation by chained equations. | | | | | | | | |

| **Supplementary Table S8. Association between last dose-to-delivery interval (days) and neonatal morbidities stratified by gestational age at birth.** | | | | | | | | |
| --- | --- | --- | --- | --- | --- | --- | --- | --- |
| **Last dose-to-delivery interval (days)** | **Gestational age at birth (weeks)** | | | | | | | |
|  | **<34** | | **34–36** | | **37–38** | | **≥39** | |
|  | **N of cases (rate)^a^** | **Adjusted OR (95 CI)^b^** | **N of cases (rate)^a^** | **Adjusted OR (95 CI)^b^** | **N of cases (rate)^a^** | **Adjusted OR (95 CI)^b^** | **N of cases (rate)^a^** | **Adjusted OR (95 CI)^b^** |
| **Any morbidity** |  |  |  |  |  |  |  |  |
| Unexposed | 38 (82.61) | Ref. | 951 (71.56) | Ref. | 11739 (54.4) | Ref. | 25929 (49.06) | Ref. |
| <2 | 25 (96.15) | 5.06 (0.62–41.26) | 144 (70.94) | 0.97 (0.69–1.36) | 14 (63.64) | 1.87 (0.84–4.17) | 0 (0) | NA |
| 2–7 | 37 (75.51) | 0.58 (0.08–4.07) | 143 (69.42) | 1.11 (0.78–1.58) | 44 (58.67) | 1.41 (0.89–2.24) | 2 (66.67) | 1.85 (0.21–16.58) |
| 8–13 | 6 (100) | NA | 46 (75.41) | 1.49 (0.79–2.81) | 40 (49.38) | 0.91 (0.57–1.44) | 0 (0) | NA |
| ≥14 | 15 (93.75) | 1.22 (0.07–20.59) | 157 (72.69) | 1.13 (0.80–1.59) | 550 (60.31) | 1.33 (1.16–1.53) | 531 (56.13) | 1.33 (1.17–1.52) |
| **Asphyxia** |  |  |  |  |  |  |  |  |
| Unexposed | 4 (8.7) | Ref. | 16 (1.2) | Ref. | 69 (0.32) | Ref. | 254 (0.48) | Ref. |
| <2 | 2 (7.69) | NA | 0 (0) | NA | 0 (0) | NA | 0 (0) | NA |
| 2–7 | 0 (0) | NA | 1 (0.49) | NA | 1 (1.33) | 4.92 (0.66–36.81) | 0 (0) | NA |
| 8–13 | 1 (16.67) | NA | 1 (1.64) | NA | 0 (0) | NA | 0 (0) | NA |
| ≥14 | 0 (0) | NA | 3 (1.39) | NA | 3 (0.33) | 1.14 (0.36–3.63) | 8 (0.84) | 1.81 (0.89–3.68) |
| **Respiratory morbidity** |  |  |  |  |  |  |  |  |
| Unexposed | 16 (34.78) | Ref. | 39 (2.93) | Ref. | 57 (0.26) | Ref. | 163 (0.31) | Ref. |
| <2 | 10 (38.46) | 1.14 (0.29–4.55) | 13 (6.4) | 2.01 (1.04–3.91) | 0 (0) | NA | 0 (0) | NA |
| 2–7 | 15 (30.61) | 0.84 (0.26–2.74) | 16 (7.77) | 2.10 (1.07–4.14) | 1 (1.33) | 4.06 (0.70–23.47) | 0 (0) | NA |
| 8–13 | 3 (50) | 6.17 (0.33–115.89) | 3 (4.92) | 1.07 (0.31–3.75) | 0 (0) | NA | 0 (0) | NA |
| ≥14 | 6 (37.5) | 0.27 (0.03–2.01) | 14 (6.48) | 1.87 (0.99–3.53) | 5 (0.55) | 1.83 (0.74–4.54) | 2 (0.21) | 0.65 (0.16–2.64) |
| **Metabolic morbidity** |  |  |  |  |  |  |  |  |
| Unexposed | 25 (54.35) | Ref. | 862 (64.86) | Ref. | 10518 (48.74) | Ref. | 22035 (41.68) | Ref. |
| <2 | 18 (69.23) | 1.21 (0.36–4.09) | 122 (60.1) | 0.81 (0.59–1.11) | 14 (63.64) | 2.43 (1.10–5.39) | 0 (0) | NA |
| 2–7 | 27 (55.1) | 0.79 (0.29–2.17) | 119 (57.77) | 0.93 (0.67–1.28) | 41 (54.67) | 1.53 (0.97–2.40) | 2 (66.67) | 2.54 (0.29–21.97) |
| 8–13 | 4 (66.67) | 4.54 (0.37–56.31) | 39 (63.93) | 1.24 (0.72–2.15) | 36 (44.44) | 0.93 (0.58–1.49) | 0 (0) | NA |
| ≥14 | 13 (81.25) | 3.13 (0.46–21.43) | 135 (62.5) | 0.96 (0.70–1.32) | 491 (53.84) | 1.29 (1.12–1.48) | 450 (47.37) | 1.26 (1.10–1.44) |
| **Infectious/Inflammatory diseases** |  |  |  |  |  |  |  |  |
| Unexposed | 21 (45.65) | Ref. | 291 (21.9) | Ref. | 3674 (17.03) | Ref. | 8945 (16.92) | Ref. |
| <2 | 14 (53.85) | 2.26 (0.56–9.07) | 51 (25.12) | 1.22 (0.86–1.75) | 2 (9.09) | 0.58 (0.14–2.41) | 0 (0) | NA |
| 2–7 | 21 (42.86) | 1.46 (0.54–3.96) | 50 (24.27) | 1.27 (0.88–1.83) | 12 (16) | 1.03 (0.56–1.91) | 0 (0) | NA |
| 8–13 | 3 (50) | 1.91 (0.20–18.20) | 16 (26.23) | 1.37 (0.75–2.49) | 14 (17.28) | 1.02 (0.57–1.81) | 0 (0) | NA |
| ≥14 | 10 (62.5) | 1.96 (0.45–8.49) | 60 (27.78) | 1.36 (0.96–1.92) | 194 (21.27) | 1.34 (1.14–1.58) | 181 (19.05) | 1.14 (0.97–1.35) |
| **Neurological morbidity** |  |  |  |  |  |  |  |  |
| Unexposed | 3 (6.52) | Ref. | 50 (3.76) | Ref. | 139 (0.64) | Ref. | 258 (0.49) | Ref. |
| <2 | 1 (3.85) | NA | 9 (4.43) | 0.99 (0.46–2.13) | 1 (4.55) | 6.49 (0.71–59.19) | 0 (0) | NA |
| 2–7 | 2 (4.08) | NA | 12 (5.83) | 1.29 (0.65–2.58) | 0 (0) | NA | 0 (0) | NA |
| 8–13 | 0 (0) | NA | 2 (3.28) | 0.71 (0.16–3.10) | 0 (0) | NA | 0 (0) | NA |
| ≥14 | 3 (18.75) | NA | 17 (7.87) | 1.86 (1.05–3.29) | 12 (1.32) | 1.78 (0.96–3.30) | 7 (0.74) | 1.35 (0.63–2.86) |
| Abbreviations: OR=Odds ratio; CI=confidence interval; NA=Not available due to convergence was not achieved due to the limited number of cases. | | | | | | | | |
| ^a^ Rate represents the number of cases per thousand infants. | | | | | | | | |
| ^b^ Model adjusted for maternal age at delivery, educational level, parity, mode of conception, pre-pregnancy BMI, hypertensive diseases, diabetic diseases, mode of delivery and vaginal bleeding during early pregnancy of the mother, as well as sex, birth weight for gestational age (percentile), and calendar year of birth of the infant, after multiple imputation by chained equations. | | | | | | | | |
